# Analyzing Hedgehog pathway-related genes: insights into breast cancer tumor microenvironment and prognostic implications

**DOI:** 10.3389/fimmu.2025.1649358

**Published:** 2025-12-01

**Authors:** Ziyun Wang, Hua Wang

**Affiliations:** Department of Breast and Thyroid Surgery, Affiliated Hospital of Nantong University, Medical School of Nantong University, Nantong, China

**Keywords:** breast cancer, Hedgehog signaling pathway, tumor microenvironment, prognostic model, immune infiltration, biomarkers

## Abstract

**Study objectives:**

Given the significant global burden of breast cancer, this study aims to systematically explore the role of Hedgehog (Hh) pathway-related genes in breast cancer prognosis and immunological characteristics. Additionally, we will construct a risk scoring system based on this pathway, with the objective of providing new references for prognostic assessment and immunotherapy strategies.

**Methods:**

Key Hh-related genes were identified, and Hedgehog scores were calculated for each sample using the ssGSEA algorithm. These scores were integrated with clinical data. Patients were classified into high-risk and low-risk groups, which were analyzed regarding immune factors, metabolic characteristics, gene mutations, and clinical outcomes.

**Results:**

Significant associations were found between the high-risk group and drug sensitivity, immune scores, and overall survival. The findings suggest that immunotherapy may serve as a valuable prognostic tool in breast cancer treatment.

**Conclusions:**

This study presents a reliable prognostic system based on the Hh pathway score for breast cancer prognosis and drug responsiveness. The results underscore the potential of immunotherapy in improving prognosis and emphasize the need for further clinical validation.

## 1 Introduction

Breast cancer is one of the most prevalent malignant tumors among women globally, representing a significant threat to public health (1). According to the latest statistics from the National Cancer Registry and Mortality Surveillance System in the United States (2), as reported by Siegel et al. (2025), breast cancer is expected to continue being the most prevalent cancer type among American women in 2025. Approximately 316,950 new cases are anticipated for the year, which will account for 32% of all new cancer cases in women (2).This epidemiological profile underscores the substantial burden of breast cancer on a global scale, highlighting the urgent need for comprehensive research into its molecular mechanisms and precision treatment strategies.

In recent years, significant advancements have been made in the diagnosis and treatment of breast cancer, particularly in molecular typing, targeted therapy, and immunotherapy (3). However, due to the high heterogeneity of tumors, challenges remain in prognosis assessment and treatment response prediction (4). Based on molecular classification, breast cancer is commonly categorized into several subtypes, including Luminal A, Luminal B, HER2-enriched, and triple-negative/basal-like, as defined by hormone receptor (ER/PR), HER2 status, and proliferative markers (such as Ki-67) (5). The tumor microenvironment (TME) plays a pivotal role in the development, progression, and treatment response of breast cancer, and the aberrant activation of various signaling pathways further complicates the disease (6).

Among these pathways, the Hedgehog (Hh) signaling pathway has attracted considerable attention for its regulatory roles in cell proliferation, differentiation, and tissue architecture (7). Existing studies indicate that abnormal activation of the Hh pathway is closely linked to the development and drug resistance of various tumors, including breast cancer (8). However, despite research identifying potential connections between the Hh pathway and breast cancer (9), the mechanisms underlying its roles in the breast cancer tumor microenvironment, immune infiltration, and prognostic prediction have yet to be systematically elucidated.

Therefore, this study aims to systematically analyze the expression characteristics and functions of Hh pathway-related genes in breast cancer, develop a prognostic prediction model based on the Hh pathway (Hedgehog score), and further investigate its potential implications for immune infiltration, drug sensitivity, and prediction of immunotherapy response. By integrating multi-omics data, this study aims to provide new molecular markers and clinical translational tools for the personalized treatment of breast cancer patients.

## 2 Materials and methods

### 2.1 Data acquisition

We obtained the TCGA BRCA dataset’s expression matrix using the TCGAbiolinks R package (10), which includes count sequencing data from 1222 breast invasive carcinoma samples. These consist of 1109 breast cancer samples and 113 adjacent non-cancerous tissue samples. The data was normalized to FPKM format for analysis. Additionally, we computed the TIDE Immunocore for patient samples using the TIDE (Tumor Immune Dysfunction and Exclusion) platform available at tide.dfci.harvard.edu (11). We accessed BRCA-related datasets (GSE7904, GSE29431, and GSE42568) from the Gene Expression Omnibus (GEO) database via the R package GEOquery (12–14).

a) GSE7904: Affymetrix Human Genome U133 Plus 2 platform includes 43 breast cancer and 19 standard tissue samples.
b) GSE29431: Gene expression profiles from 54 breast cancer patients and 12 normal tissue samples.
c) GSE42568: Affymetrix Human Genome U133 Plus 2.0 Array includes 104 breast cancer and 17 standard tissue samples.

We acquired 50 immune checkpoint genes from cited literature (15), precisely designated in **Supplementary Table S2**. We retrieved 32 HLA family genes from the GeneCards database using “Human Leukocyte Antigen” as the search term. Subsequently, we retained genes whose Gene Symbol commenced with “HLA,” as detailed in **Tables 1** and **2**. Somatic mutation data, including single-nucleotide (16) variations (SNVs), were obtained from the TCGA-BRCA dataset via the TCGA website. We visualized this data using the R package maftools. Copy Number Variation (CNV) data from the same dataset were procured using the TCGAbiolinks R package and analyzed with GISTIC 2.0 (17). We employed the cBioPortal database (18),accessed through http://www.cbioportal.org, to obtain data on microsatellite instability (MSI) and tumor mutation burden (TMB). The TIDE score was calculated using the TIDE algorithm from the TIDE website. Given patient privacy and data-sharing policies for the dataset, we used publicly available data from databases such as TCGA (The Cancer Genome Atlas) and GEO (Gene Expression Omnibus). To facilitate further access for researchers, we provide detailed instructions for data download and preprocessing on jianguoyun, along with specific guidelines on obtaining these data from public databases.

**Table 1:** BRCA dataset information list.

Ontology	ID	Description	GeneRatio	BgRatio	pvalue	p.adjust
BP	GO:0007224	smoothened signaling pathway	40/205	140/18800	<0.001	<0.001
BP	GO:0010498	proteasomal protein catabolic process	54/205	496/18800	<0.001	<0.001
BP	GO:0008589	regulation of smoothened signaling pathway	27/205	82/18800	<0.001	<0.001
BP	GO:0007389	pattern specification process	39/205	463/18800	<0.001	<0.001
BP	GO:0016055	Wnt signaling pathway	37/205	452/18800	<0.001	<0.001
CC	GO:0000502	proteasome complex	41/206	59/19594	<0.001	<0.001
CC	GO:1905369	endopeptidase complex	41/206	79/19594	<0.001	<0.001
CC	GO:1905368	peptidase complex	41/206	111/19594	<0.001	<0.001
CC	GO:0022624	proteasome accessory complex	20/206	23/19594	<0.001	<0.001
CC	GO:0005839	proteasome core complex	18/206	20/19594	<0.001	<0.001
MF	GO:0004298	threonine-type endopeptidase activity	11/202	14/18410	<0.001	<0.001
MF	GO:0001664	G protein-coupled receptor binding	26/202	288/18410	<0.001	<0.001
MF	GO:0070003	threonine-type peptidase activity	11/202	24/18410	<0.001	<0.001
MF	GO:0005109	frizzled binding	12/202	38/18410	<0.001	<0.001
MF	GO:0005125	cytokine activity	22/202	235/18410	<0.001	<0.001

HRDEGs, Hedgehog - related differentially expressed genes; GO, Gene Ontology; BP, biological process; CC, cellular component; MF, molecular function.

**Table 2:** KEGG enrichment analysis results of HRDEGs.

Ontology	ID	Description	GeneRatio	BgRatio	pvalue	p.adjust
KEGG	hsa03050	Proteasome	39/175	46/8164	<0.001	<0.001
KEGG	hsa04340	Hedgehog signaling pathway	40/175	56/8164	<0.001	<0.001
KEGG	hsa05010	Alzheimer disease	62/175	384/8164	<0.001	<0.001
KEGG	hsa05022	Pathways of neurodegeneration - multiple diseases	62/175	476/8164	<0.001	<0.001
KEGG	hsa05012	Parkinson disease	48/175	266/8164	<0.001	<0.001

HRDEGs, Hedgehog-related differentially expressed genes; KEGG, Kyoto Encyclopedia of Genes and Genomes.

### 2.2 Sample inclusion and exclusion criteria

In this study, we employed the following criteria for sample inclusion and exclusion:

*Inclusion Criteria*:

Patients diagnosed with breast invasive carcinoma (BRCA), Availability of surgical tissue samples for analysis, Written informed consent was obtained from each patient.

*Exclusion Criteria*:

Patients with incomplete clinical data, Prior treatments that may influence tumor characteristics, and the Presence of other concurrent malignancies.

### 2.3 Data standardization

Normalization was performed using the limma R package, and dataset distribution was visualized via boxplots. Differentially expressed genes (DEGs) with logFC < 0 and p-value < 0.05 were identified and compared with Hedgehog-related genes (HRGs) using Venn diagrams, volcano plots, and differential rank maps generated with ggplot2.

### 2.4 Standardization of breast cancer datasets

**Figure 1:**
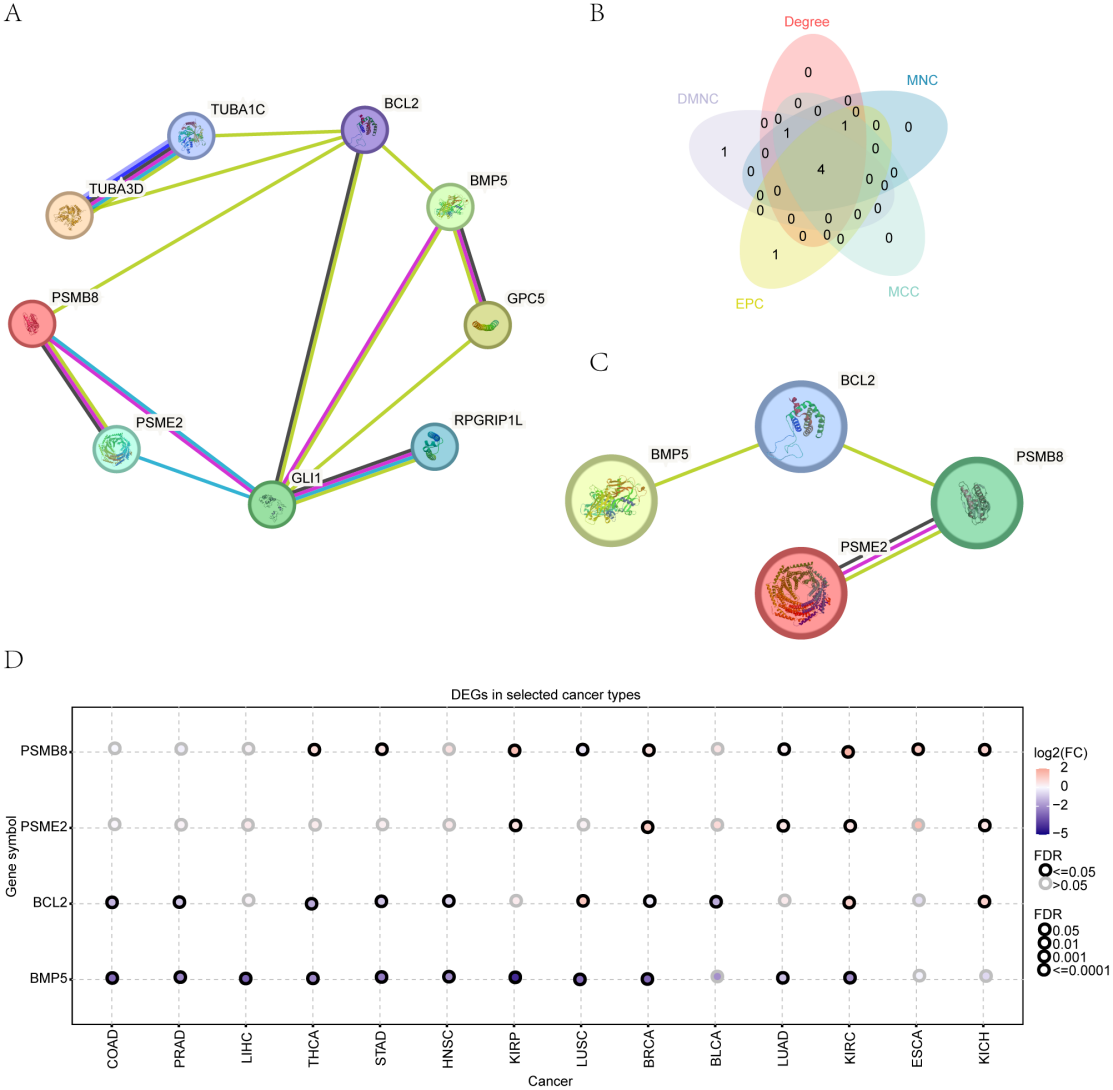
Protein-protein interaction network. **(A)** Network visualization of protein-protein interactions among HRDEGs. **(B)** Identification of top hub genes using five different algorithms. **(C)** Gene expression patterns of key HRDEGs across 14 cancer types. **(D)** Expression patterns of 4 key genes in 14 types of cancer.

First, we standardized the breast cancer datasets GSE7904, GSE29431 and GSE42568 using the R package limma package (**Figures 1A–F**). According to the figure, the dataset GSE7904 (**Figures 1A, B**), The expression profile data of GSE29431 (**Figures 1C, D**) and GSE42568 (**Figures 1E, F**) tended to be consistent among the samples after normalization.

### 2.5 Single-cell RNA analysis

To delineate the tumor immune microenvironment at single-cell resolution, we utilized the curated dataset GSE114725 from the GEO database, which comprises 23,688 single cells profiled using the 10x Genomics Chromium platform. Data preprocessing, quality control, and normalization were conducted using Cell Ranger and the Seurat R package, adhering to established best practices. Subsequent clustering analysis facilitated the identification of major cellular subsetsrcationntt tumor epithelial cells, T cells, B cells, macrophages, endothelial cells, and fibroblasts,onntt on canonical markers (e.g., EPCAM, CD3D, MS4A1, CD68, PECAM1, ACTA2). Cell type annotations were further validated against reference datasets such as the Human Primary Cell Atlas to ensure accuracy and reproducibility. Within this refined cellular landscape, we specifically examined the expression patterns of PSMB8 and BCL2 across immune cell subtypes relevant to breast cancer, leveraging the analytical framework provided by the IMMUcan database (18).We performed functional enrichment analysis using Gene Ontology (GO) and pathway enrichment analysis with the Kyoto Encyclopedia of Genes and Genomes (KEGG) for insights into gene functions at varying expression levels. Gene Ontology (GO) facilitates large-scale functional enrichment investigations, encompassing biological processes (BP), molecular function (MF), and cellular components (CC) (19).The KEGG database contains valuable information on genomes, biological pathways, diseases, and medications (20, 21). We ensured statistical significance using clusterProfiler (an R package) for GO annotation analysis of Hedgehog-Related Differentially Expressed Genes (HRDEGs), setting a significance threshold at p < 0.05 and a False Discovery Rate (FDR) value (q-value) below 0.05 (22).

### 2.6 Gene Set Enrichment and Variation Analysis (GSEA & GSVA)

We performed Gene Set Enrichment Analysis (GSEA) using the clusterProfiler software, prioritizing genes based on log fold change (logFC) and identifying enriched gene sets from the MSigDB database using the “c2.cp.v7.2.symbols.gmt” set. Significant enrichment was defined by a p-value < 0.05 and false discovery rate (FDR) < 0.25, which strikes a balance between biological discovery and error control (23). Additionally, we applied Gene Set Variation Analysis (GSVA), an unsupervised method independent of parameter settings. GSVA was conducted using the “h.all.v7.4.symbols.gmt” gene set from MSigDB on the TCGA-BRCA dataset. This allowed us to assess variations in functional enrichment between normal and BRCA patient samples (24).

### 2.7 Cox prognostic screening

To construct a prognostic model for Hedgehog-Related Genes (HRGs) in the Hh pathway and evaluate their predictive potential, we used univariate Cox regression analysis on the BRCA breast cancer dataset, combining Overall Survival (OS) and time. Genes with a p-value < 0.01 were further analyzed using multivariate Cox regression to develop the model and calculate RiskScore. Adopting a stricter p-value standard can help narrow the range of candidate genes or features, reduce the risk of false positives, and provide a more reliable basis for experimental validation and clinical application. The RiskScore, also referred to as the Hedgehog score, was constructed based on nine prognosis-related genes: TUBA3D, TUBA1C, PSME2, RPGRIP1L, PSMB8, BCL2, GLI1, GPC5, and BMP5. These genes were selected through multifactorial Cox regression analysis without any reduction in their number.


RiskScore=TUBA3D*(−0.0108)+TUBA1C*(0.0145)+PSME2*(−0.0147)+RPGRIP1L*(0.1664)+PSMB8*(−0.0032)+BCL2*(−0.0088)+GLI1*(−0.3239)+GPC5*(−0.1818)+BMP5*(0.0007)

### 2.8 Protein-protein interaction

We used the STRING database to construct a protein-protein interaction network for differentially expressed genes, setting a minimum interaction score of 0.150 (25). The cytoHubba plugin, employing DEGREE Correlation, MNC, MCC, EPC, and DMNC algorithms, identified the top six HRDEGs (26). A correlation heatmap visualized connections between immune checkpoint genes, HLA family genes, and key genes. Access the starBase database (version 3.0) at https://rnasysu.com/chipbase3/index.php and the CHIPBase database (version 3.0) at https://discover.nci.nih.gov/cellminer/home.do (27, 28).We used the hTFtarget database to identify transcription factors interacting with essential genes (29).

### 2.9 Analyses of CNV, somatic mutation, and functional similarity

We analyzed CNV, somatic mutations (SNPs), and functional similarity using TCGAbiolinks, GISTIC 2.0, VarScan, and maftools (30). The functional similarity was assessed using the GOSemSim R package.

### 2.10 Immune infiltration analysis

We assessed the prevalence and abundance of immune-infiltrating cells using single-sample Gene Set Enrichment Analysis (ssGSEA) through the GSVA package in R. Enrichment scores were calculated to quantify immune cell infiltration across various categories (31). Additionally, following previously established guidelines (32), we estimated the presence of eight immune cell types and two non-immune stromal cell groups within the tumor microenvironment. Spearman’s correlation analysis evaluated the statistical relationship between MCPcounter abundance estimates, antigen gene expression, and immune infiltration, with statistical significance set at p-value < 0.05.

### 2.11 Sensitivity analysis of key genes

This section details an evaluation designed to determine how the expression stability of key genes influences the variation of other variables within our dataset. The GDSC database (cancerRxgene.org) contains extensive information on molecular markers associated with drug sensitivity and response in cancer cells. The CCLE repository (https://portals.broadinstitute.org/ccle/about) Genomic alterations in cancer significantly influence treatment response and often serve as biomarkers for drug therapy (33). The GDSC database (cancerRxgene.org) contains extensive information on molecular markers associated with drug sensitivity and response in cancer cells. The CCLE repository. The CellMiner database (https://discover.nci.nih.gov/cellminer/home) offers experimental data and vital genes for the initial dataset (34). These tools facilitate the comprehensive investigation of specific genes, cells, or test data, allowing for the storage, retrieval, and download of molecular profiling data for various cancer cell types. We analyzed drug sensitivity for essential genes using expression levels and drug information from the GDSC, CCLE, and CellMiner databases.

### 2.12 BRCA disease subtype identification

The Consensus Clustering algorithm uses resampling to determine the number of subgroups and evaluate clustering reliability. This technique aids in understanding cluster stability and parameter determination (35). To categorize distinct BRCA subtypes in the TCGA-BRCA dataset, we used the ConsensusClusterPlus R package, generating clusters ranging from 2 to 8 and conducting 50 iterations while randomly selecting 80% of the samples. The clustering algorithm was “km” with the “euclidean” distance metric.

### 2.13 Calculation of Hedgehog score

Using ssGSEA, we determined the relative prevalence of individual genes in each dataset sample. Hs values for TCGA-BRCA samples were computed using the ssGSEA algorithm from the GSVA R package. We divided the BRCA samples into Hs_high and Hs-low groups based on the median Hs. Tumor purity was evaluated using the ESTIMATE algorithm, calculating immune and stromal scores from the expression data.

### 2.14 Immunotherapy

To investigate the predictive significance of Hedgehog scores (Hs) about immunotherapy, we obtained Hs data from the TCIA database (https://www.tcia.at/home) (36). Using the ggplot function in R, we generated a boxplot illustrating the differences between Hs high and low clusters of TCGA-BRCA patients across various Immune Phenotype Scores (IPS). We conducted a multivariate Cox regression analysis using TCGA-BRCA expression profile data, incorporating Hs and clinicopathological factors, to create a clinical prediction nomogram with the rms package in R. Calibration charts compared projected values of the nomogram with actual survival rates.

### 2.15 Real-time quantitative PCR

We collected 271 tissue samples from individuals who underwent surgical removal, with ethics committee approval from Nantong University (protocol code: 2020-L125) and informed consent from participants. RNA extraction from paraffin-embedded tissues was performed using a Thermo Fisher Scientific kit. qRT-PCR assessed mRNA levels of PSMB8, BCL2, BMP5, and PSME2, using GAPDH as the internal control. Primer sequences were as follows:

- GAPDH (forward): 5’-TGGCCATTAGGACCGAGACTT-3’
- GAPDH (reverse): 5’-CACCCTGTTGCTGTAGCCAAA-3’
- PSMB8 (forward): 5’-CAGTGTCGGCAGCCTCCAAG-3’
- PSMB8 (reverse): 5’-GACCCTTCTTATCCCAGCCACAG-3’
- BCL2 (forward): 5’-GGCACCTGCACACCTGGATC-3’
- BCL2 (reverse): 5’-TTCCCCGCATCCCACTCGTAG-3’
- BMP5 (forward): 5’-GGCATCCTTGGCAGAAGAGACC-3’
- BMP5 (reverse): 5’-TCATGGAGGCTGGCTAGAGGAG-3’
- PSME2 (forward): 5’-AGAAAGTCCTGTCCCTGCTTGC-3’
- PSME2 (reverse): 5’-CACCTTCTCCTGGATTGCTACCC-3’.

### 2.15 Statistical analysis

All data processing and analyses were conducted using R software (version 4.1.2). We first applied the Shapiro-Wilk test for each continuous variable to determine normal distribution, with a P-value below 0.05 indicating a non-normal distribution. An independent t-test assessed the statistical significance of constant variables for normally distributed data. At the same time, the Mann–Whitney U test (Wilcoxon rank-sum test) was used for non-normally distributed data. Categorical variables were analyzed using the chi-square or Fisher’s exact test, and the Kruskal–Wallis test compared three or more groups. Kaplan–Meier survival curves, generated using the R survival package, illustrated survival variations, with the Log-rank test determining significance. Statistical significance was defined as a P-value below 0.05; all P-values were two-sided.

## 3 Results

### 3.1 Analysis of differentially expressed genes in breast cancer

**Figure 2:**
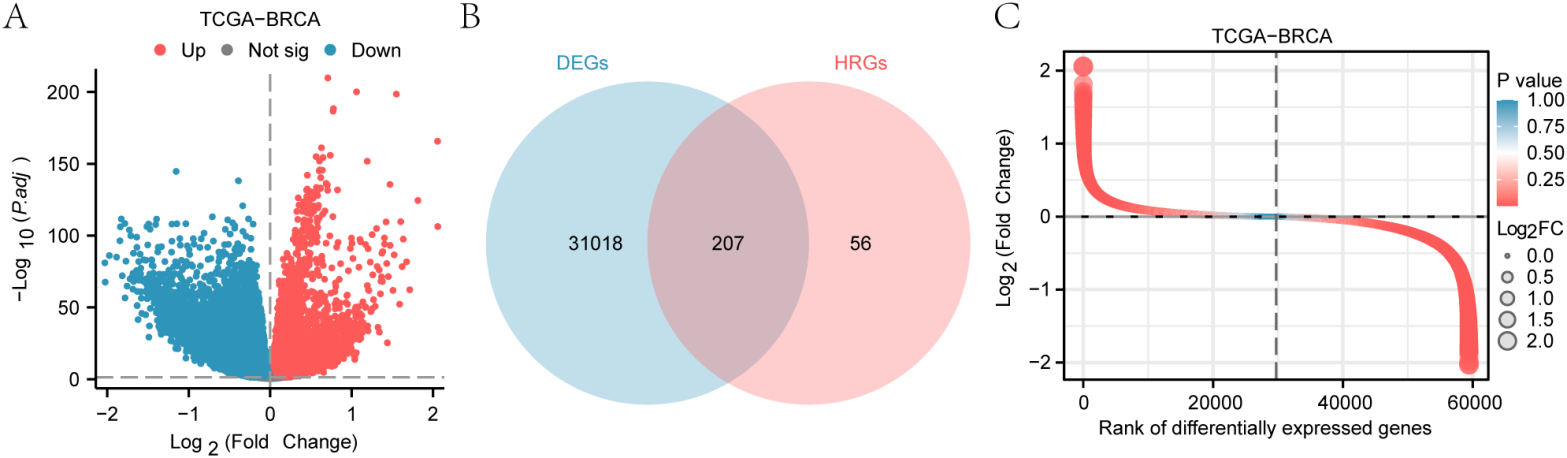
Analysis of differentially expressed genes in the TCGA-BRCA dataset. **(A)** Volcano plot of differentially expressed genes (DEGs) in breast cancer samples. **(B)** Venn diagram illustrating the overlap between DEGs and Hedgehog-related genes (HRGs). **(C)** Heatmap depicting the expression levels of Hedgehog-related differentially expressed genes (HRDEGs) across BRCA and normal tissue samples.

Using the limma package, we analyzed the TCGA-BRCA dataset and identified 31,255 differentially expressed genes (DEGs) (**Table 3**), with 12,683 upregulated and 18,542 downregulated in breast cancer (BRCA) samples (**Figure 2A**).

**Table 3:** BRCA dataset information list.

Dataset Characteristic	TCGA-BRCA	GSE7904	GSE29431	GSE42568
Platform		GPL570	GPL570	GPL570
Species	Homo sapiens	Homo sapiens	Homo sapiens	Homo sapiens
Samples in the Normal group	113	19	12	17
Samples in the BRCA group	1109	43	54	104

TCGA: the cancer genome atlas: BRCA: breast invasive carcinoma

To isolate Hedgehog-related DEGs (HRDEGs), we filtered for DEGs and Hedgehog-related genes (HRGs), identifying 207 HRDEGs (**Figure 2B**). A heatmap shows the expression of these HRDEGs across BRCA and normal samples (**Figure 2C**).

### 3.2 Enrichment analysis of Hedgehog-related differentially expressed genes in breast cancer

We performed enrichment analysis on 207 HRDEGs using GO and KEGG, with significant results (P < 0.05, FDR < 0.05) visualized in bubble charts, circular network diagrams, and bar graphs (**Supplementary Figure S12**). Key GO enrichments included Wnt, Notch, transcription regulation pathways, and components like the proteasome and peptidase complexes. KEGG analysis highlighted JAK-STAT, TGF-beta, Hedgehog, Wnt, and apoptosis pathways, providing insights into gene interactions and breast cancer progression.

In the context of GO and KEGG enrichment, significant items were selected based on their p-values and false discovery rate (FDR) values, with a threshold set at < 0.05. This analysis helps us understand the functional relevance and potential pathways associated with the Highly Differentially Expressed Genes, shedding light on their role in breast cancer development and progression.

### 3.3 Utilization of GSEA and GSVA techniques for enrichment analysis

We conducted Gene Set Enrichment Analysis (GSEA) and Gene Set Variation Analysis (GSVA) on the TCGA-BRCA dataset to explore the relationship between gene expression levels and associated biological processes. Gene Set Enrichment Analysis (GSEA): GSEA identified significant enrichments in the TP53, MAPK, Notch, Wnt, and Hedgehog pathways (**Supplementary Figures S13A–F**), suggesting their potential association with breast cancer development and progression. The enrichment results indicate the statistical clustering tendencies of the relevant gene sets; however, they do not directly imply that these pathways are necessarily in a higher activation state at the functional level. Detailed results are summarized in **Table 4**.

**Table 4:** GSEA analysis of TCGA-BRCA.

ID	setSize	enrichmentScore	NES	P value	p. adjust	Q value
REACTOME_CELL_CYCLE_CHECKPOINTS	29<0.001	0.605	2.407	<0.001	<0.001	<0.001
REACTOME_CHROMOSOME_MAINTENANCE	139.000	0.624	2.315	<0.001	<0.001	<0.001
REACTOME_TRNA_PROCESSING	108.000	0.641	2.296	<0.001	<0.001	<0.001
REACTOME_HOMOLOGY_DIRECTED_REPAIR	137.000	0.620	2.292	<0.001	<0.001	<0.001
REACTOME_G2_M_CHECKPOINTS	166.000	0.584	2.224	<0.001	<0.001	<0.001
REACTOME_RRNA_PROCESSING	203.000	0.565	2.186	<0.001	<0.001	<0.001
REACTOME_DNA_REPLICATION	185.000	0.564	2.155	<0.001	<0.001	<0.001
REACTOME_DNA_DOUBLE_STRAND_BREAK_REPAIR	167.000	0.557	2.117	<0.001	<0.001	<0.001
REACTOME_PROCESSING_OF_CAPPED_INTRON_CONTAINING_PRE_MRNA	244.000	0.537	2.103	<0.001	<0.001	<0.001
REACTOME_DNA_REPAIR	332.000	0.515	2.076	<0.001	<0.001	<0.001

TCGA, the cancer genome atlas: BRCA, breast invasive carcinoma; GSEA, Gene Set Enrichment Analysis.

Gene Set Variation Analysis (GSVA): GSVA provided insights into differences between breast cancer samples (BRCA group) and standard samples (Normal group). This analysis identified significant variations in gene sets, particularly in the Notch and Wnt pathways (**Supplementary Figure S12G**). Further details are provided in, **Table 5**.

**Table 5:** GSVA analysis of TCGA-BRCA.

Pathway Name	logFC	AveExpr	t	P.Value	adj.P.Val
HALLMARK_UV_RESPONSE_DN	0.449	-0.017	15.969	<0.001	<0.001
HALLMARK_MYOGENESIS	0.302	0.011	13.086	<0.001	<0.001
HALLMARK_WNT_BETA_CATENIN_SIGNALING	0.347	-0.024	12.748	<0.001	<0.001
HALLMARK_HEDGEHOG_SIGNALING	0.342	0.012	12.234	<0.001	<0.001
HALLMARK_BILE_ACID_METABOLISM	0.246	-0.002	11.510	<0.001	<0.001
HALLMARK_KRAS_SIGNALING_UP	0.284	0.010	10.820	<0.001	<0.001
HALLMARK_APICAL_JUNCTION	0.257	0.002	10.770	<0.001	<0.001
HALLMARK_TNFA_SIGNALING_VIA_NFKB	0.309	-0.012	10.254	<0.001	<0.001
HALLMARK_HEME_METABOLISM	0.193	-0.029	9.752	<0.001	<0.001
HALLMARK_KRAS_SIGNALING_DN	0.156	0.049	9.709	<0.001	<0.001
HALLMARK_ADIPOGENESIS	0.242	-0.029	9.560	<0.001	<0.001
HALLMARK_HYPOXIA	0.224	-0.021	9.128	<0.001	<0.001
HALLMARK_TGF_BETA_SIGNALING	0.217	-0.022	7.244	<0.001	<0.001
HALLMARK_XENOBIOTIC_METABOLISM	0.136	0.002	6.398	<0.001	<0.001
HALLMARK_PANCREAS_BETA_CELLS	0.142	0.074	6.286	<0.001	<0.001

TCGA, the cancer genome atlas; BRCA, breast invasive carcinoma; GSVA, Gene Set Variation Analysis.

Overall, GSEA and GSVA elucidate gene expression’s impact on breast cancer and identify critical pathways involved in its progression.

### 3.4 Cox prognostic screening and risk model development

**Figure 3:**
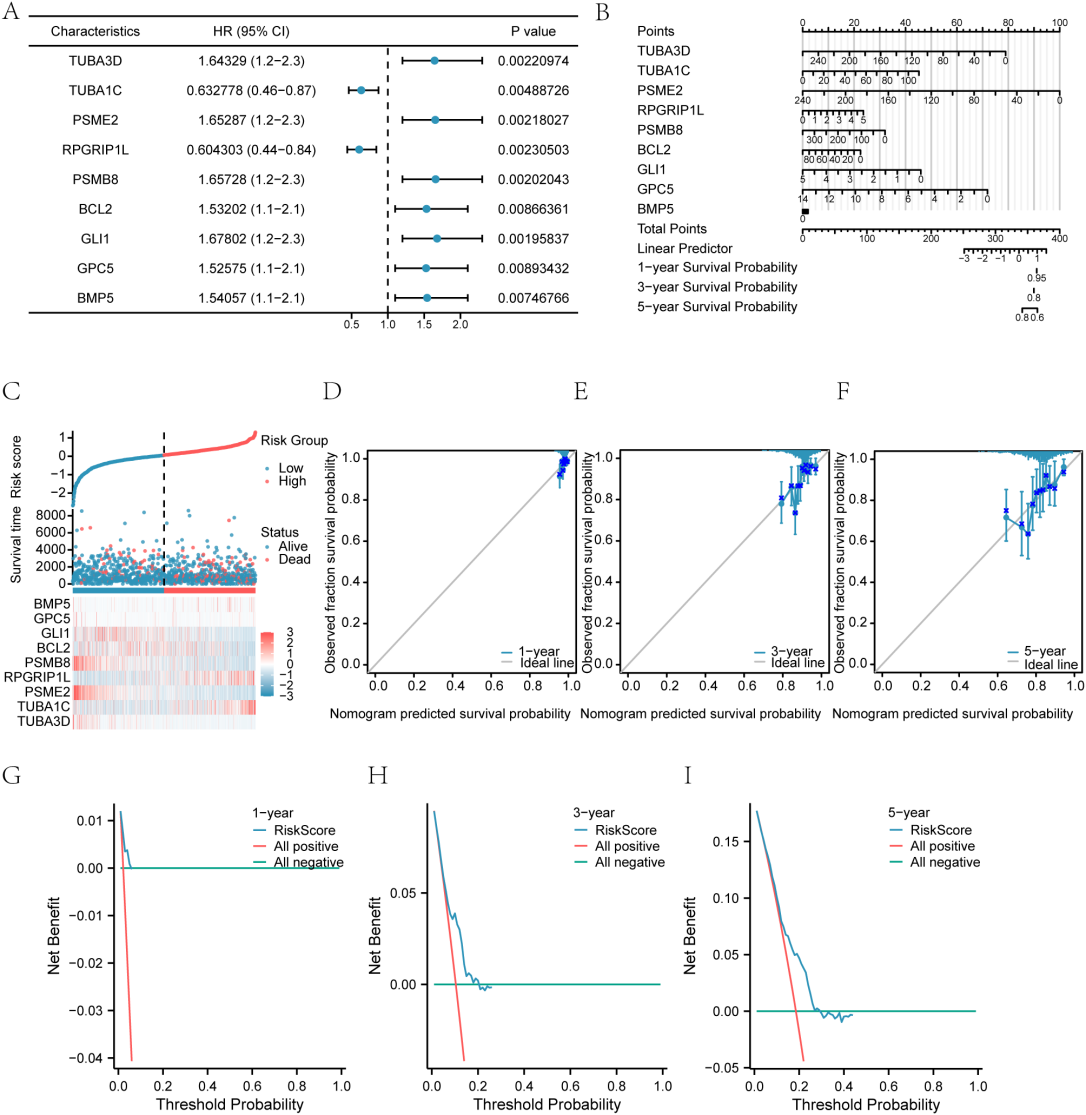
Cox prognostic model and risk stratification. **(A)** Univariate Cox regression identifying significant Hedgehog-related genes (HRGs) for risk stratification in breast cancer. **(B)** Nomogram predicting 1-, 3-, and 5-year survival outcomes for patients based on the prognostic model. **(C)** Distribution of patient risk scores. **(D)** Survival status scatter plot versus the ranked risk score. **(E)** Heatmap illustrates the expression patterns of signature HRGs across the risk groups. **(F)** Kaplan-Meier curves compare overall survival between the high- and low-risk groups. **(G)** Time-dependent ROC curves assess the model's predictive accuracy for 1-, 3-, and 5-year survival. **(H)** Principal component analysis (PCA) visualizes the separation of samples based on risk group. **(I)** Decision curve analysis (DCA) evaluates the net clinical benefit of the model at 1, 3, and 5 years.

To develop a predictive model for highly differentially expressed genes (HRDEGs) associated with the Hedgehog (Hh) pathway, we analyzed overall survival (OS) data from the TCGA-BRCA breast cancer dataset. Univariate Cox regression identified nine significant HRDEGs (TUBA3D, TUBA1C, PSME2, RPGRIP1L, PSMB8, BCL2, GLI1, GPC5, and BMP5) with p-values < 0.01 (**Figure 3A**).

These genes were incorporated into a multivariate Cox regression model, creating a risk score to categorize patients into high- and low-risk groups (**Table 6**). The model’s predictive ability was assessed using a nomogram (**Figure 3B**), indicating that PSME2 had the highest diagnostic efficacy while BMP5 had the lowest.

**Table 6:** Cox regression to identify clinical features of dataset TCGA-BRCA.

Characteristics	HR Multivariate analysis	CI Low Multivariate analysis	CI High Multivariate analysis	P value Multivariate analysis
TUBA3D	0.989	0.970	1.009	0.287
TUBA1C	1.015	1.001	1.028	0.032
PSME2	0.985	0.974	0.997	0.016
RPGRIP1L	1.181	0.926	1.506	0.179
PSMB8	0.997	0.988	1.005	0.451
BCL2	0.991	0.976	1.007	0.263
GLI1	0.723	0.471	1.112	0.140
GPC5	0.834	0.440	1.581	0.578
BMP5	1.001	0.974	1.028	0.962

TCGA, the cancer genome atlas; BRCA, breast invasive carcinoma; CI, confidence interval.

The nomogram was adapted for 1-, 3-, and 5-year predictions (**Figures 3D–F**), with calibration curves confirming alignment with actual survival outcomes. Decision Curve Analysis (DCA) demonstrated that the 1-year model offered greater clinical utility than the 3- and 5-year models (**Figures 3G–I**).

We also generated OS Kaplan-Meier (KM) curves using the risk scores from the TCGA-BRCA dataset (**Supplementary Figure S2A**). This analysis revealed that higher risk scores significantly predicted poorer OS (P < 0.001), as illustrated in **Supplementary Figure S2B**. Time-dependent AUC curves for 1, 3, and 5 years showed the RiskScore’s effectiveness, with AUCs of 0.7–0.9 for 1 and 3 years, while the 5-year prediction had lower accuracy (AUC 0.5–0.7).

In summary, the time-sensitive ROC curves (1, 3, and 5 years) for the RiskScore (**Figure 3**) demonstrated moderate accuracy for the first two years and relatively poor prediction for the fifth year.

### 3.5 Protein-protein interaction network

The interaction relationships among the genes were visually represented using Cytoscape software (**Figure 1A**). We employed the cytoHubba plug-in to analyze five algorithms (MCC, DMNC, EPC, Degree, and Closeness), identifying the top four HRDEGs—PSMB8, BCL2, BMP5, and PSME2—as essential genes (**Figures 1B, C**). Using the GSCA online tool, we examined the expression patterns of these genes across 14 cancer types, which required a minimum of three tumor samples alongside their normal counterparts (**Figure 1D** and **Table 1**). The results indicated a consistent decrease in BCL2 and BMP5 expression in most tumor samples, while PSMB8 and PSME2 showed increased expression.

### 3.6 Constructing mRNA-RBP and mRNA-TF interaction networks

**Figure 4:**
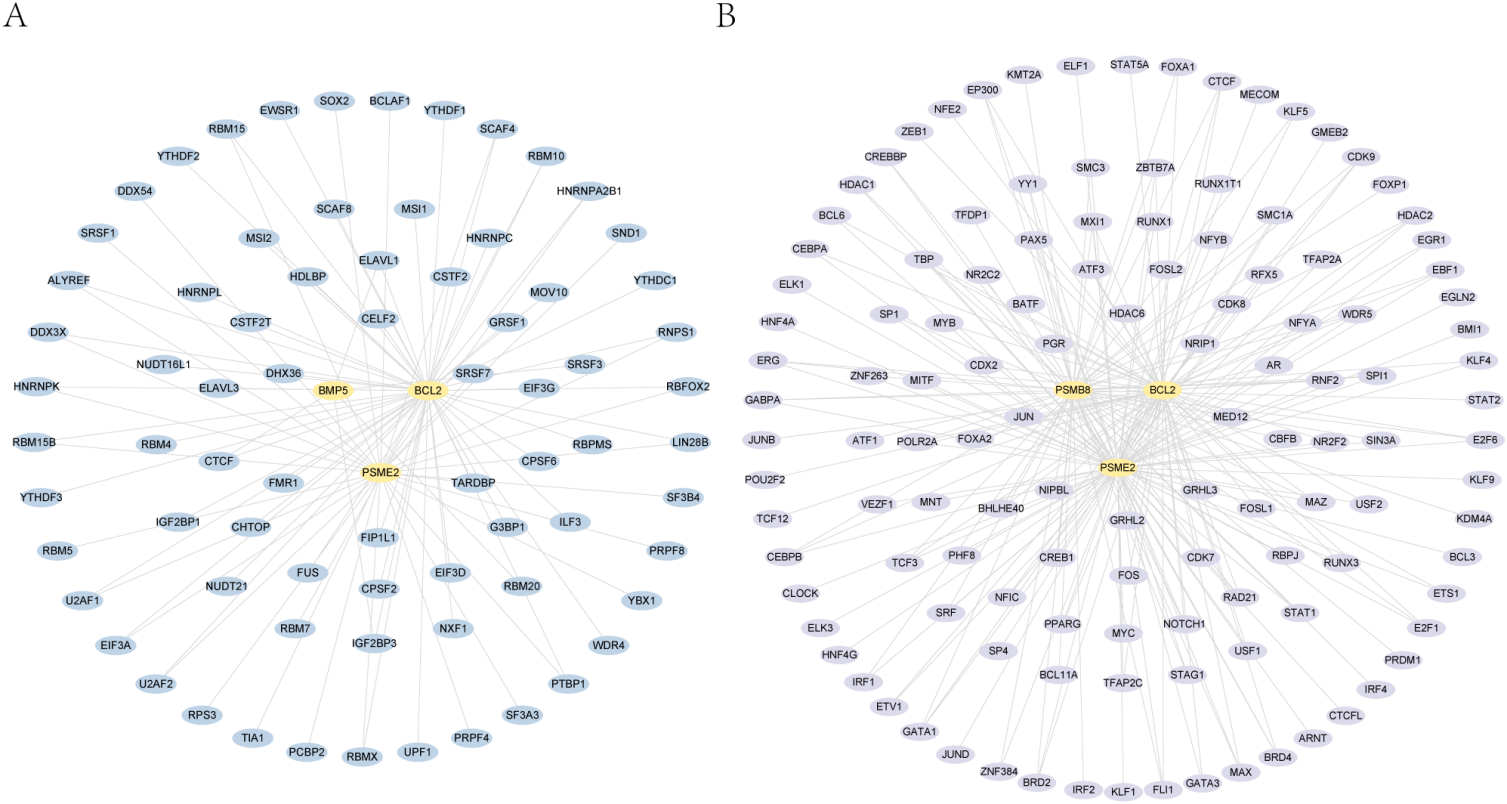
mRNA-RBP and mRNA-TF interaction networks. This figure shows the RNA-binding proteins (RBPs) and transcription factors (TFs) interacting with four essential genes: PSMB8, BCL2, BMP5, and PSME2. **A.** key gene-RBP interaction network. **B.** key gene-transcription factor interaction network.

We utilized the ENCORI database to predict interactions between RNA-binding proteins (RBPs) and four essential genes: PSMB8, BCL2, BMP5, and PSME2. The interaction network shows connections between BCL2, BMP5, and PSME2 with 74 RBPs, totaling 94 connections (**Figure 4A**; **Table 2**).

We also analyzed the CHIPBase (version 3.0) and target databases to identify transcription factors (TFs) interacting with these genes. The network reveals interactions between PSMB8, BCL2, and PSME2 with 124 TFs, resulting in 211 connections (**Figure 4B**; **Supplementary Table 5**).

### 3.7 Expression analysis of key genes in the TCGA-BRCA breast cancer dataset

**Figure 5:**
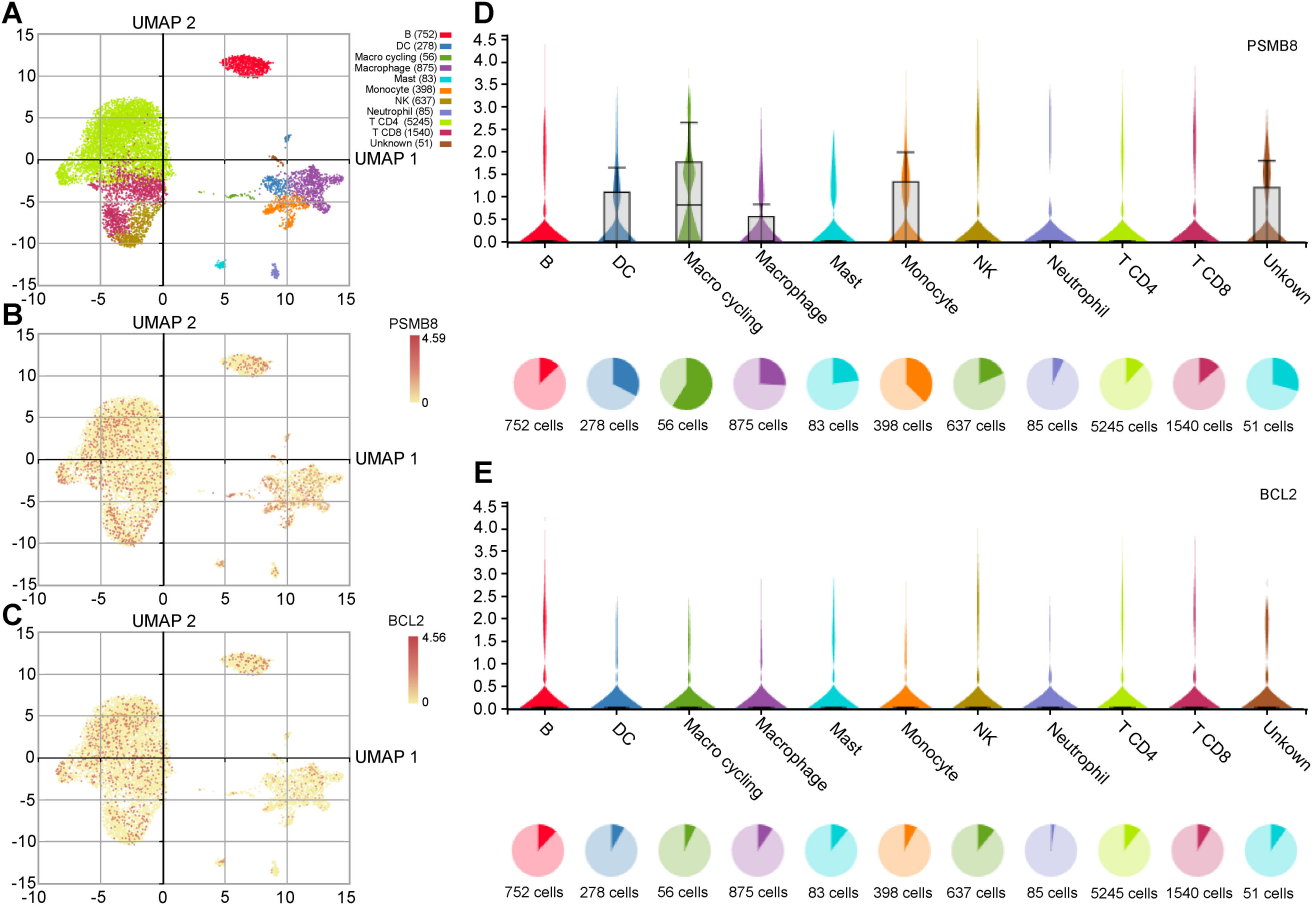
Expression analysis of key genes in immune cells. **(A)** UMAP plot of immune cells in the breast cancer microenvironment. **(B, C)** UMAP distribution diagram shows the relative expression of PSMB8 and BCL2 in each cell. **(D, E)** Violin plots show the relative expression levels of PSMB8 and BCL2 in 8 types of cells.

We used the IMMUcan database to analyze single-cell RNA data and examine the expression of PSMB8, BCL2, and PSME2 in the breast cancer immune microenvironment. **Figure 5A** shows the annotation of various immune cell types in breast cancer. PSMB8, BCL2, and PSME2 were expressed in tumor cells, stromal cells, and immune cell subsets (**Figures 5B, C**). PSMB8 was primarily expressed in macrophages, BCL2 in B cells, and PSME2 in monocytes (**Figures 5D, E**).

The TCGA-BRCA dataset demonstrated distinct accuracies for BMP5, PSMB8, PSME2, and BCL2, with varying AUC values across categories.

**Figure 6:**
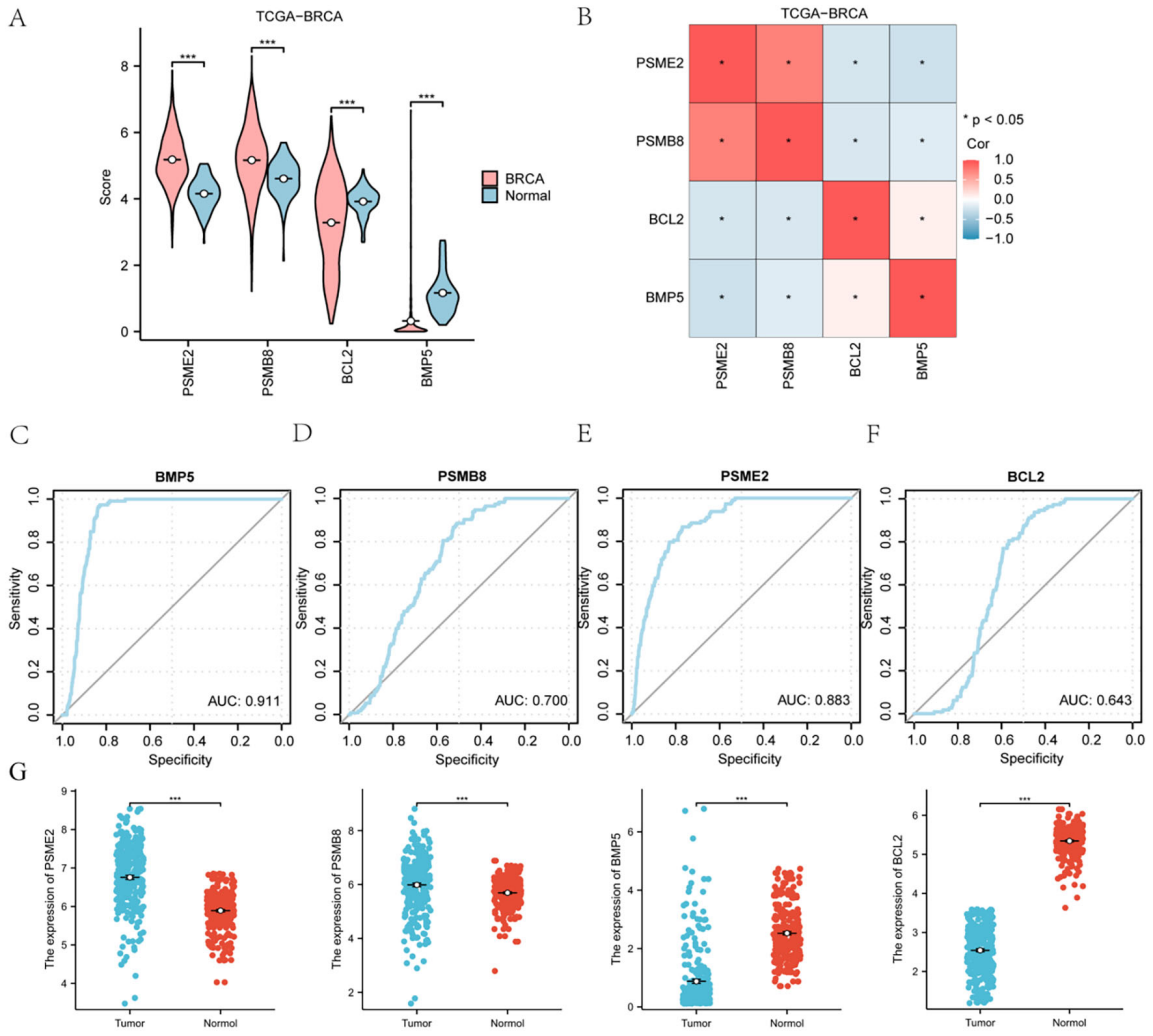
Expression of Key Genes in the TCGA-BRCA Breast Cancer Dataset: Bubble charts **(A, B)**, circular network diagrams **(C, D)**, and bar graphs **(E, F)** illustrating significant Gene Ontology (GO) terms and Kyoto Encyclopedia of Genes and Genomes (KEGG) pathways associated with HRDEGs in breast cancer. Key pathways include Wnt, Notch, JAK-STAT, and TGF-beta, highlighting their roles in cancer progression. **(G)** qPCR results from clinical samples corroborate the cancer-specific expression patterns of the four genes. *P < 0.05, **P < 0.01, ***P < 0.001.

This figure compares the expression of key genes, including BMP5, PSMB8, PSME2, and BCL2, between BRCA and normal groups, where red represents BRCA and blue represents normal. A heat map visualizes the correlation among these significant genes within the TCGA-BRCA dataset. ROC curve analysis was conducted for BMP5, PSMB8, PSME2, and BCL2, assessing their diagnostic performance. The area under the curve (AUC) values indicates varying levels of precision: values above 0.9 suggest high precision, values between 0.7 and 0.9 indicate moderate precision, and values between 0.5 and 0.7 reflect relatively low precision. Additionally, PCR results reveal that PSME2 and PSMB8 are highly expressed in cancer tissues, while BMP5 and BCL2 are more highly expressed in normal tissues (**Figures 6A–C**).

### 3.8 Expression analysis of essential genes in the GEO dataset

We analyzed the expression of four essential genes (PSMB8, BCL2, BMP5, PSME2) using the GSE7904 dataset (**Figure 4A**) and examined their differential expression between BRCA and normal groups in GSE29431 (**Figure 4B**) and GSE42568 (**Figure 4C**). After excluding genes not present in these datasets, we focused on PSMB8, BCL2, and BMP5. In GSE7904, PSMB8 and BCL2 showed significant differences between BRCA and normal groups (p < 0.05). In GSE29431, BMP5 (p < 0.001) and BCL2 (p < 0.05) exhibited significant differences, and in GSE42568, both BMP5 and BCL2 (p < 0.001) and PSMB8 (p < 0.01) were significantly different. ROC curves for the three genes in these datasets were plotted, excluding results with AUC values below 0.6. In GSE7904, PSMB8 and BCL2 had low accuracy (AUC 0.5–0.7). BMP5 and BCL2 in GSE29431 and BMP5, BCL2, and PSMB8 in GSE42568 demonstrated moderate accuracy (AUC 0.7–0.9).

### 3.9 Prognostic performance

**Figure 7:**
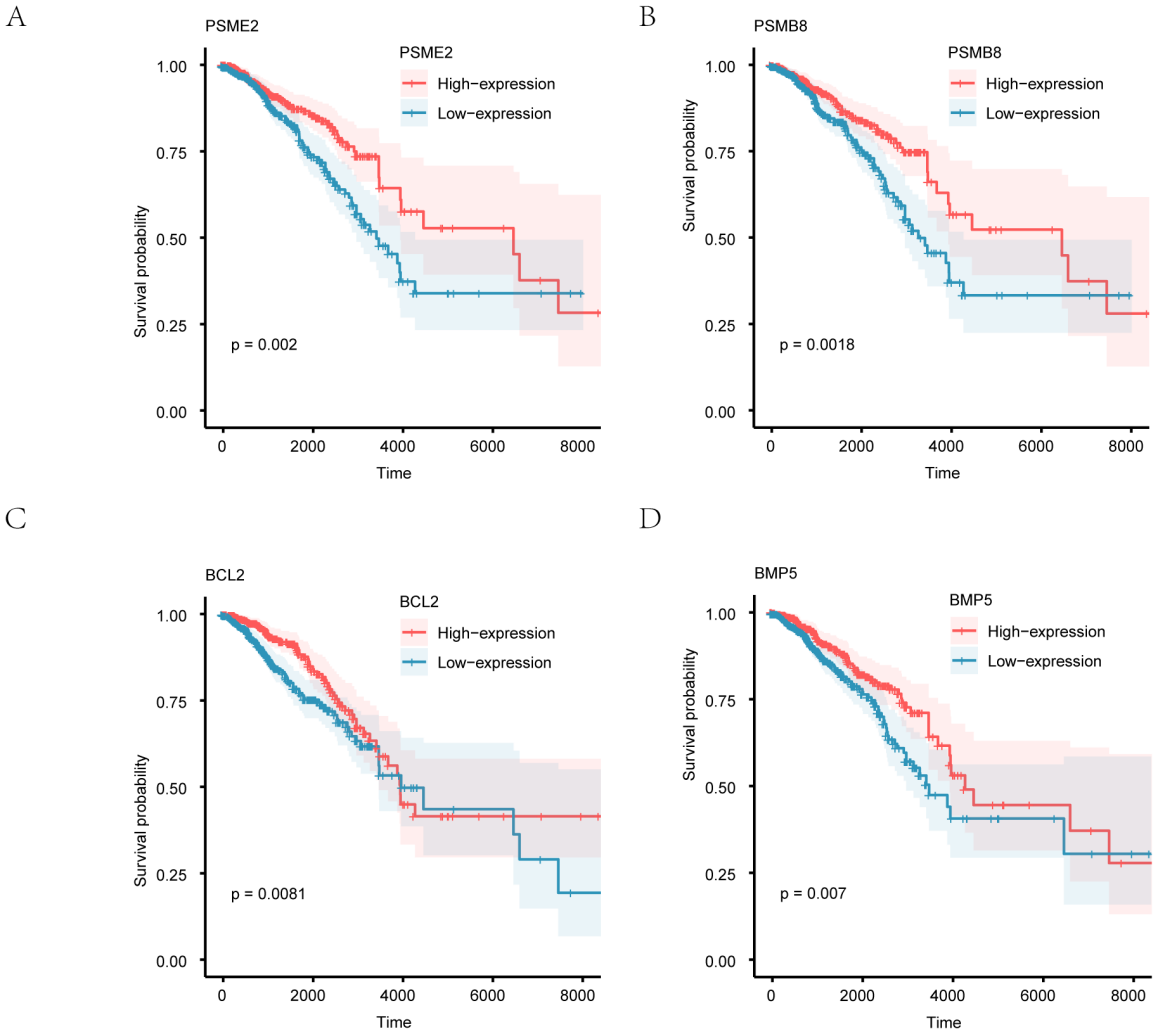
depicts the prognostic analysis of key genes using Kaplan–Meier (KM) curves for overall survival (OS). Kaplan–Meier curves depict the association between gene expression levels and overall survival for PSME2 **(A)**, PSMB8 **(B)**, BCL2 **(C)**, and BMP5 **(D)**. Patients were stratified into high-expression (red) and low-expression (blue) groups based on the median expression level of each gene.

The TCGA-BRCA breast cancer dataset exhibits separate Kaplan–Meier survival curves (KM) for four notable genes (PSMB8, BCL2, BMP5, and PSME2). Statistical significance was set below 0.05, indicating that the four crucial genes in the TCGA-BRCA breast cancer dataset were statistically significant (**Figures 7A–D**).

**Figure 8:**
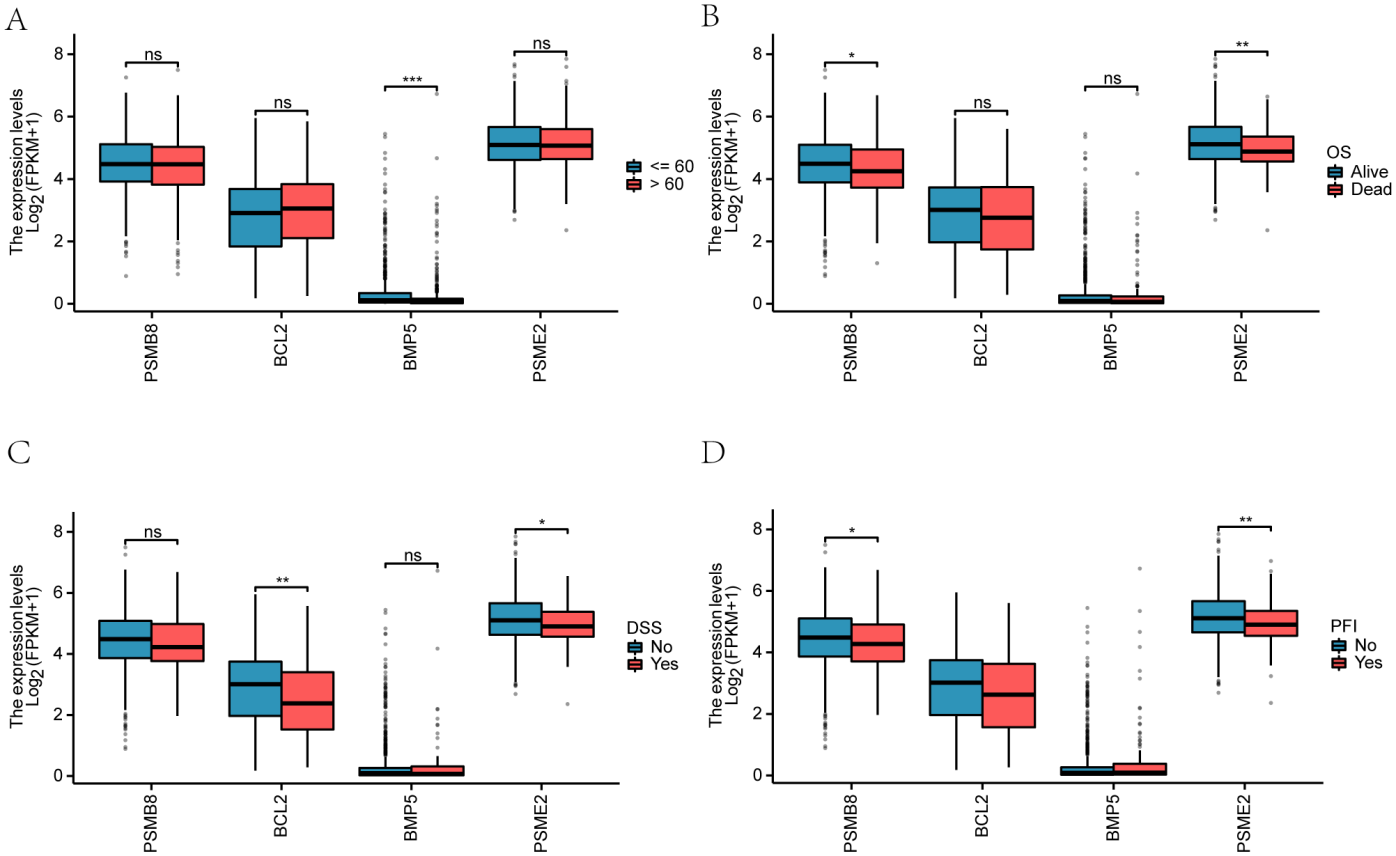
Prognostic analysis of key genes. **(A-D)** Kaplan-Meier survival curves for PSMB8, BCL2, BMP5, and PSME2, showing overall survival differences between high and low expression groups.

**Figure 8** depicts the prognostic analysis of key genes using Kaplan–Meier (KM) curves for overall survival (OS). The KM curves for PSME2 (A), PSMB8 (B), BCL2 (C), and BMP5 (D) were examined for their prognostic value. In these curves, the Low-expression group of breast cancer patient samples is represented in blue, while the High-expression group is shown in red. Notable distinctions are indicated by P < 0.05, while differences with a high significance level are marked as P < 0.01.

### 3.10 Clinical correlation of key genes

**Figure 9:**
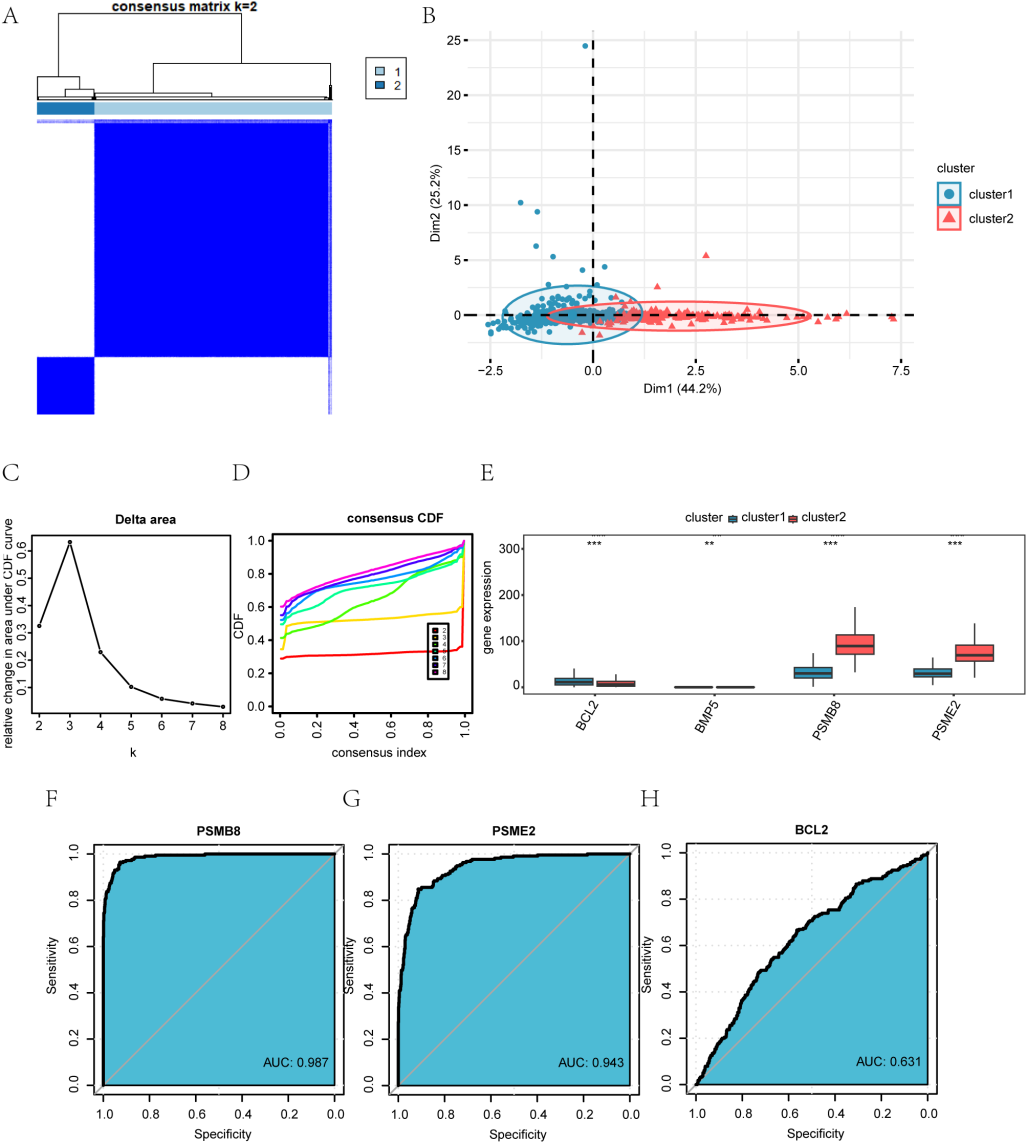
Identification and characterization of BRCA-associated disease subtypes. **(A)** Consensus clustering matrix for k = 2 in the TCGA-BRCA dataset. **(B)** PCA plot showing the separation of the two disease subtypes (Cluster 1 and Cluster 2). **(C)** Delta area plot indicating the relative change in consensus distribution function (CDF) area for different cluster numbers (k). **(D)** Cumulative distribution function (CDF) curves for different k values. **(E)** Expression levels of the four key genes across the identified subtypes. **(F–H)** Receiver operating characteristic (ROC) curves evaluating the discriminatory power of PSMB8 **(F)**, PSME2 **(G)**, and BCL2 **(H)** between Cluster 1 and Cluster 2.*P < 0.05, **P < 0.01, ***P < 0.001.

We examined the clinical correlation of four key genes (PSMB8, BCL2, BMP5, and PSME2) with breast cancer prognosis. A violin plot (**Figure 9A**) showed gene expression variations by age (≤65 and >65) in the TCGA-BRCA dataset, with BMP5 demonstrating a significant difference (p < 0.001). We also analyzed the relationship between gene expression and clinicopathological factors, including disease-specific survival (DSS) and progression-free interval (PFI) (**Supplementary Figures 8B-D**).

Overall survival (OS) analysis revealed significant differences (p < 0.01), particularly for PSMB8, which differed notably between Alive and Dead groups (p < 0.05). BCL2 showed significant variation in DSS (p < 0.01), while PSME2 significantly influenced both DSS (p < 0.05) and PFI (p < 0.01). Additionally, PSMB8 exhibited significant variations in PFI between the Alive and Dead groups (p < 0.05).

- (A) Violin plot comparing key gene outcomes by age groups (≤65 in blue, >65 in red).
- (B-D) Comparisons of key genes among Alive and Dead groups regarding OS, DSS, and PFI.

### 3.11 Analyses of CNV, SNP, and functional similarity (friends)

A comprehensive analysis of copy number variations (CNVs) in the TCGA-BRCA cohort, utilizing GISTIC 2.0, identified four key genesifiediv BCL2, BMP5, and PSME2ified recurrent genomic alterations. Among these, PSMB8 exhibited the highest frequency of amplifications, while BCL2 demonstrated the most frequent deletions (**Supplementary Figure S5A**). Notably, PSME2 displayed a substantial number of both amplification and deletion events (**Supplementary Figure S5A**), indicating underlying genomic instability at this locus. Somatic mutation profiling conducted using the maftools R package further delineated nine major mutation categories, with missense mutations being predominant. Single nucleotide variants (SNVs), particularly C-to-T transitions, were the most frequently observed (**Supplementary Figure S5B**). Functional similarity (Friends) analysis underscored a potentially central role for PSME2 in breast cancer pathogenesis, as it approached the threshold of statistical significance (**Supplementary Figure S5C**). Consistent with this finding, subsequent immune infiltration and survival analyses revealed significant associations between PSME2 alterations and the levels of various tumor-infiltrating immune cells, as well as overall survival (OS). These results suggest that CNV aberrations in PSME2 may contribute to breast tumorigenesis by modulating gene expression and shaping the tumor immune microenvironment.

### 3.12 Immune infiltration analysis (ssGSEA and MCPCounter)

We applied the ssGSEA method to assess the relationship between the expression profiles of 28 immune cells in the TCGA-BRCA dataset across different categories (Normal/BRCA). Boxplots (**Supplementary Figure 6A**) revealed significant differences (p < 0.05) in the expression of 21 immune cell types, including activated B cells, CD4 T cells, CD8 T cells, dendritic cells, and natural killer cells. A heatmap (**Supplementary Figure 6B**) showed the correlation between the expression of four key genes (PSMB8, BCL2, BMP5, PSME2) and immune cell infiltration, with significant associations (p < 0.05). PSMB8 correlated positively with CD56dim NK cells and PSME2 with Type 1 Th cells, while negative correlations were observed for PSME2 with plasmacytoid dendritic cells and BCL2 with CD56bright NK cells. Using the MCPCounter algorithm, we identified 10 distinct immune cell categories in the TCGA-BRCA dataset, with significant correlations, such as PSMB8 with T cells/CD8 T cells and inverse relationships between PSME2 and endothelial cells and BCL2 with monocytic lineage cells (**Supplementary Figure 6C**).

### 3.13 Drug sensitivity analysis

This section presents a drug sensitivity analysis using data from the CellMiner, GDSC, and CCLE databases. We developed a ridge regression model using the prophetic algorithm to predict gene responsiveness to anticancer drugs based on IC50 measurements by integrating mRNA expression profiles with drug activity data for key genes. In the CellMiner database, BCL2 was associated with 6-chloro-2-methoxy-9-[(5-piperidinopentyl)amino]acridine and enhydrin A, while PSMB8 correlated with gw772405x and negatively with zimelidine hydrochloride (**Supplementary Figure 7A**). The GDSC database showed positive correlations between BCL2, Tanespimycin, and BMP5 with T0901317, though BCL2 had negative correlations with most small drug molecules (**Supplementary Figure 7B**). In the CCLE database, BCL2 was inversely correlated with L-685458 and panobinostat (**Supplementary Figure 7C**).

### 3.14 Examining the association between immune checkpoints and genes in the HLA family

We identified 29 immune checkpoint genes by intersecting a set of 50 known genes with the TCGA-BRCA dataset. We compared the expression levels of four key genes (PSMB8, BCL2, BMP5, and PSME2) with these 29 immune checkpoint genes (**Supplementary Figure 8A**). Notably, PSMB8 showed a significant correlation with CD27 (R = 0.502, p-value = 0).

In addition, we integrated 19 genes from the HLA family by merging 32 HLA genes with the TCGA-BRCA dataset. Our correlation analysis revealed specific relationships between the four main genes and HLA genes (**Supplementary Figure S8B**). For instance, PSMB8 strongly correlated with HLA-A (R = 0.812, p-value = 0), while PSME2 moderately correlated with HLA-E and HLA-A (R = 0.514).

- B: PCA analysis results for the two breast cancer subtypes (Cluster 1 and Cluster 2) in the TCGA-BRCA dataset.
- C-D: Delta plot of the area under the CDF curve for various cluster numbers (C), and the cumulative distribution function (CDF) plot for consistency clusters (D).
- E: Group comparison of the four key genes across different breast cancer subtypes in the TCGA-BRCA dataset.
- F-H: ROC curves for PSMB8 (F), PSME2 (G), and BCL2 (H) across the different subtypes of breast cancer (Cluster 1 and Cluster 2) in the TCGA-BRCA dataset.

### 3.15 Construction of BRCA-associated disease subtypes

Using the ‘ConsensusClusterPlus’ R package, we analyzed the expression of four key genes (PSMB8, BCL2, BMP5, PSME2) in BRCA samples from the TCGA-BRCA dataset. Consensus clustering identified two BRCA subtypes: Cluster 1 (894 samples) and Cluster 2 (215 samples) (**Figure 8A**). Principal Component Analysis (PCA) revealed significant differences between these subtypes (**Figure 8B**). The delta chart and CDF diagram confirmed optimal clustering with two clusters (k=2) (**Supplementary Figures 12C, D**). Expression levels of PSMB8, BCL2, and PSME2 differed significantly (P < 0.001) between clusters, while BMP5 showed significant differences (P < 0.01) (**Figure 8E**). A Kaplan-Meier curve was created based on the overall survival (OS) and OS time data (**Supplementary Figure S1**). ROC curves showed that PSMB8 and PSME2 had high precision (AUC > 0.9) in distinguishing BRCA subtypes, while BCL2 had limited precision (AUC 0.5-0.7) (**Supplementary Figures 12F–H**).

### 3.16 Hedgehog pathway score

#### 3.16.1 Analysis of Hedgehog pathway score in breast cancer

**Figure 10:**
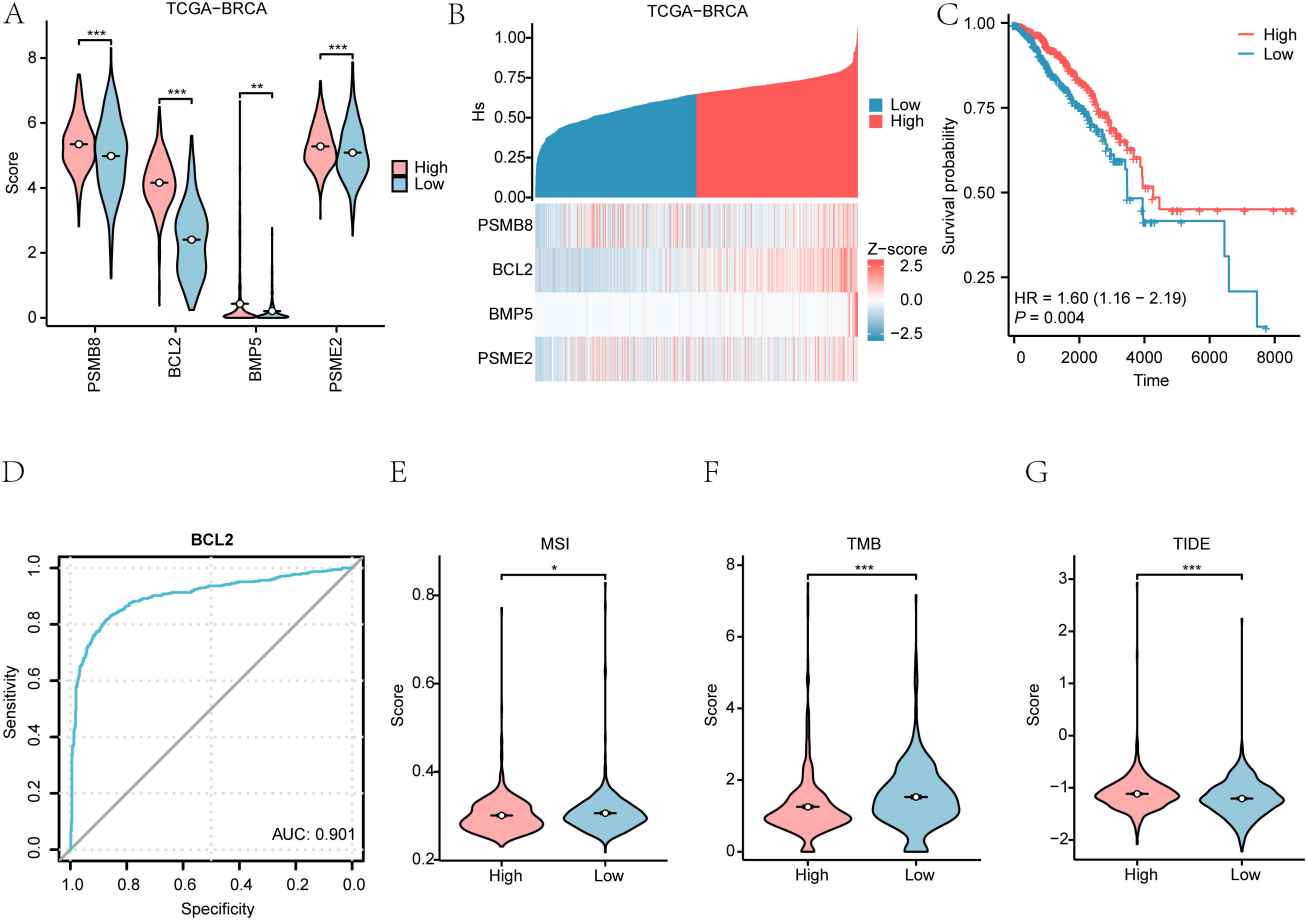
Hedgehog pathway score in breast cancer. **(A)** Differences in Hedgehog pathway scores (Hs) between high and low expression groups. **(B)** Correlation of Hedgehog pathway scores with gene expression. **(C)** Kaplan-Meier survival curves based on Hs scores. **(D)** ROC curve of the key gene BCL2 between High/Low Hs groups in the dataset TCGA-BRCA disease patient samples. **(E-G)** Group comparison plots of MSI **(E)**, TMB **(F)**, TIDE score **(G)** in the High/Low risk groups of Hs are shown. *P < 0.05, **P < 0.01, ***P < 0.001.

We explored the Hedgehog pathway score (Hs) in breast cancer patients using the ssGSEA algorithm on the TCGA-BRCA dataset. The Hs was calculated based on the expression levels of four essential genes: PSMB8, BCL2, BMP5, and PSME2. The results illustrated significant differences in gene expression between patients with high and low Hs (p < 0.001) (**Figure 10A**). Further analysis assessed the relationships among these four genes (**Figure 10B**).

#### 3.16.2 Survival outcomes based on Hedgehog pathway score

Kaplan-Meier plots demonstrated a significant difference in overall survival (OS) outcomes based on Hs (p < 0.01) (**Figure 10C**). ROC curves indicated that BCL2 had substantial accuracy (AUC > 0.9) in distinguishing between high and low Hs groups (**Figure 10D**).

#### 3.16.3 Evaluation of microsatellite instability and tumor mutation burden

**Figure 11:**
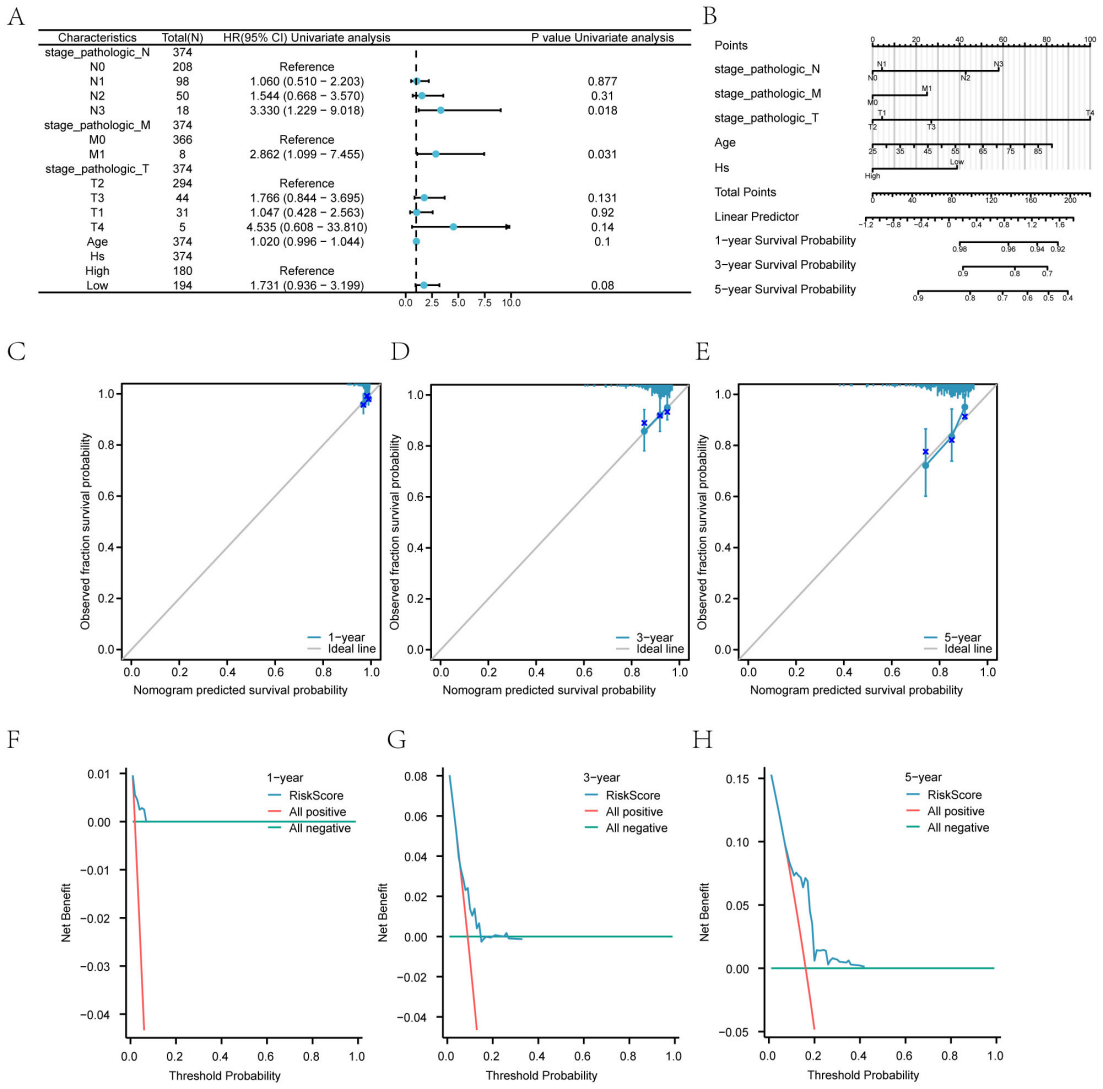
Prognostic Model and Clinical Correlations **(A)** Forest plot of COX regression for Hs. **(B)** Nomograms of clinical prediction models. **(C-E)** Calibration curves of the clinical prediction model at 1 year (**Supplementary Figure S12C**), 3 years (**Supplementary Figure S12D**), and 5 years (**Supplementary Figure S12E**). **(F-H)** DCA plots of the clinical prediction model at 1 year (**Supplementary Figure S12F**), 3 years (20G), and 5 years (**Supplementary Figure S12H**). Hs: Hedgehog score.

**Figure 12:**
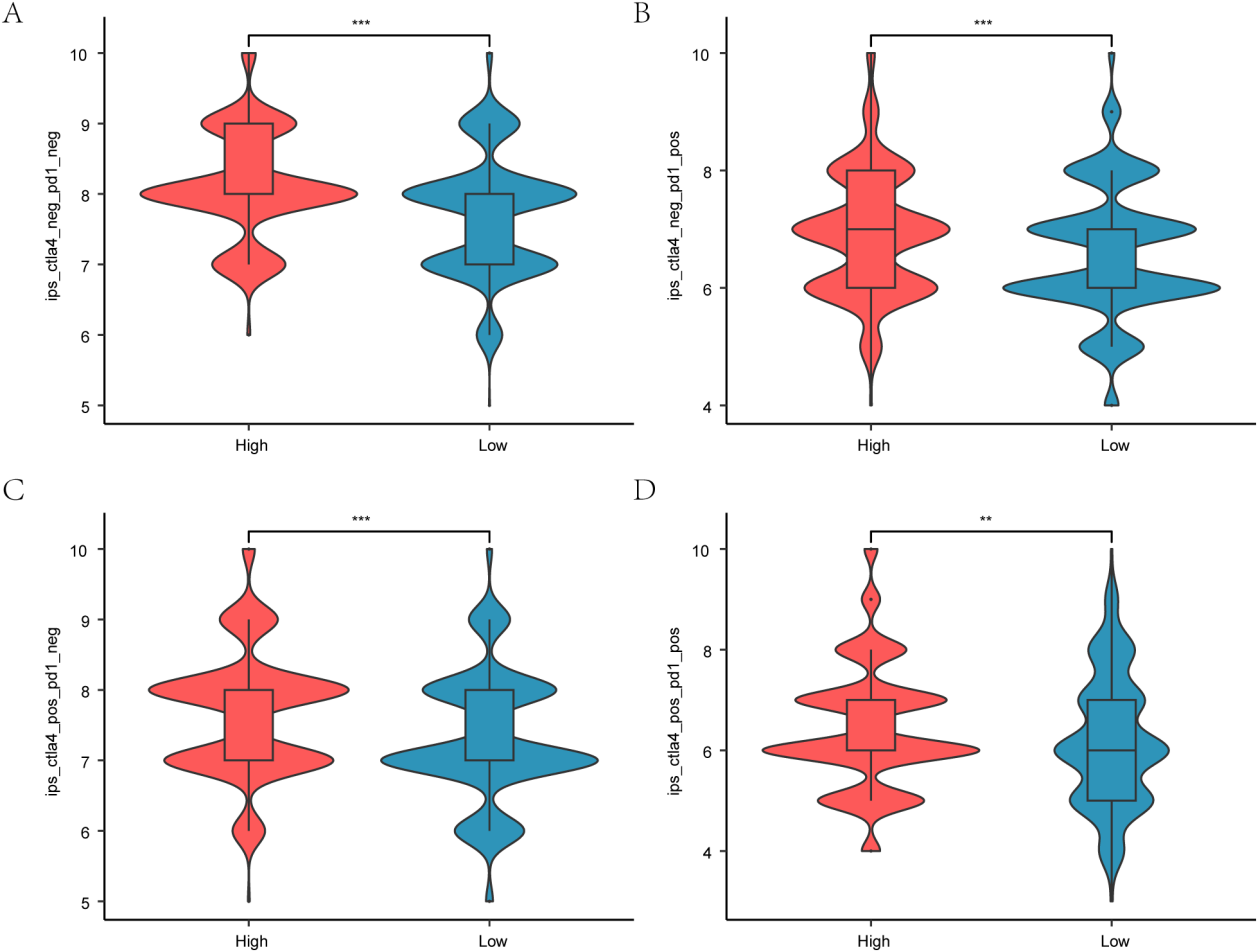
Immunotherapy and Hedgehog pathway score. **A.** boxplot comparing Hedgehog pathway scores with immune phenotype scores in breast cancer patients, showing predictive potential for immunotherapy. **B.** Boxplot of IPS-PD1/PD-L1/PD-L2 differences between different subtypes in TCIA-BRCA. **C.** Boxplot of IPS-CTLA4 differences between different subtypes in TCIA-BRCA. **D.** Boxplot of different subtypes of IPS-PD1/PD-L1/PD-L2 + CTLA4 in TCIA-BRCA.*P < 0.05, **P < 0.01, ***P < 0.001.

We evaluated microsatellite instability (MSI) and tumor mutation burden (TMB) in breast cancer patients, revealing significant differences between high and low-risk Hs groups (p < 0.05) (**Figures 11E–G**). The immunotherapy analysis utilizing the Hs model can be observed in **Figure 12**.

### 3.17 Prognostic model and clinical correlation of Hedgehog pathway score

In the TCGA-BRCA dataset, we developed a prognostic model based on the Hs score. A forest plot (**Supplementary Figure 2A**) summarizes the results of the Cox regression analysis for Hs. A predictive chart combining Hs, age, and stage was created to assess breast cancer patients’ overall survival (OS) (**Supplementary Figure 2B**).

#### 3.17.1 Calibration and decision curve analysis

The calibration graph illustrates high accuracy at 1, 3, and 5-year intervals, showing strong concordance between estimated and observed OS values (**Supplementary Figures 12C–E**). The practical utility of the Hs prediction model was evaluated through Decision Curve Analysis (DCA) for one year (**Supplementary Figure 12F**), three years (**Supplementary Figure 12G**), and five years (**Supplementary Figure 12H**). The analysis indicates that the 5-year model offers greater clinical value than the 3-year model, with the 1-year model demonstrating the highest practicality.

### 3.18 Enrichment analysis using the Hs model for GSEA

We performed a GSEA enrichment analysis on the TCGA-BRCA dataset to assess gene expression impacts on breast cancer across high and low Hs score groups (**Figure 8A**). This analysis revealed significant associations with various biological pathways, including Cornified Envelope formation (**Figure 8B**), Keratinization (**Figure 8C**), Fcgr Activation (**Figure 8D**), and Fatty Acid Metabolism (**Figure 8F**), among others (**Table 6**).

### 3.18 Somatic mutation analysis

This section examines genetic alterations, focusing on single nucleotide polymorphisms (SNPs) in the TCGA-BRCA dataset. Using R and map tools, mutation analysis identified nine primary types of somatic alterations, with missense mutations being the most common (**Supplementary Figure S10**). SNPs, particularly C to-T transitions, were predominant (**Figures 10A, C**). PIK3CA had the highest mutation rate in the high HR score group (38%), while TP53 accounted for 51% of mutations in the low HR score group (**Figures 10B, D**).

### 3.19 Drug sensitivity analysis

We evaluated drug sensitivity using GDSC database data to predict responses of breast cancer patients with varying HR scores to anti-tumor medications. The Mann–Whitney U test identified significant differences in sensitivity to the top 20 drugs (**Figure 12**). Notably, distinct sensitivity profiles emerged for these medications between high- and low-HR score categories, indicating substantial disparities.

## 4 Discussion

In our analysis of differential gene expression within the TCGA-BRCA dataset using the limma package, we identified 31,255 genes that met the criteria of logFC > 0 and P-value < 0.05. Specifically, 12,683 genes exhibited increased expression in the breast cancer (BRCA) group compared to normal tissue, while 18,542 showed decreased expression. This extensive differential expression highlights the complexity of breast cancer pathology and underscores the potential for targeted therapeutic strategies.

We focused on the differentially expressed genes associated with the Hedgehog signaling pathway (HRDEGs), identifying a total of 207 HRDEGs for functional enrichment analysis. Our key findings highlight the Wnt and Notch signaling pathways, which may play significant roles in the progression of breast cancer and in the development of treatment resistance. This aligns with existing literature emphasizing the importance of these pathways as potential therapeutic targets (37–39).

GO and KEGG enrichment analyses revealed biological processes and pathways, such as apoptosis and cell signaling, that may play significant roles in the development of breast cancer. Identifying the JAK-STAT and TGF-beta pathways suggests that targeting these routes may improve treatment outcomes by addressing immune evasion mechanisms commonly observed in tumors (40). These findings are consistent with prior studies documenting similar pathways in breast cancer biology (41).

Our Gene Set Enrichment Analysis (GSEA) and Gene Set Variation Analysis (GSVA) further elucidate the potential relationships between gene expression levels and their biological significance across various breast cancer subtypes. The significant enrichment of the TP53, MAPK, and Wnt pathways suggests possible associations between these pathways and tumor aggressiveness, as well as patient prognosis. These associations provide a theoretical basis for utilizing these pathways as biomarkers for disease stratification.

We constructed a predictive model based on Hedgehog pathway-related differentially expressed genes (HRDEGs) through univariate and multivariate Cox regression analyses. This model effectively stratifies patients into high-risk and low-risk groups based on their overall survival. The risk score holds significant prognostic value, further enhancing the clinical relevance of our findings and supporting the integration of genomic data into clinical practice. However, this study has certain limitations. Due to constraints in sample size, completeness of clinical information, and data accessibility, we were unable to perform multivariate analyses based on biological subtypes, staging, and other critical clinicopathological features. The absence of key clinical variables in the current public databases, coupled with insufficient sample sizes after stratification, makes it challenging to conduct effective multivariate regression modeling for patients with different subtypes or stages. This limitation affects the model’s generalizability and interpretability across various biological subtypes or clinical stratifications. Such constraints should be fully considered when inferring the extrapolability of research conclusions. Future efforts should focus on integrating more multicenter samples with complete clinical information, particularly those encompassing multidimensional variables such as molecular subtypes, stages, and treatment regimens, to refine multivariate regression analysis strategies. This will enhance the model’s applicability, scientific rigor, and clinical reference value. Additionally, it is recommended to further elucidate the specific mechanisms of this pathway and its key genes in breast cancer through protein level detection, functional intervention experiments, and validation with independent clinical samples.

Our identification of PSME2 as a gene with high diagnostic efficacy mirrors findings from other studies highlighting its role in cancer biology (42). Conversely, BMP5’s lower diagnostic efficacy raises questions about its functional role in breast cancer, necessitating further investigation into its mechanistic pathways (43).

The interaction network analysis revealed significant RNA-binding protein (RBP) and transcription factor (TF) associations with key genes, particularly PSMB8, BCL2, and PSME2. Understanding these interactions may provide insights into gene expression regulation and tumor microenvironment dynamics, which are critical for breast cancer progression. Targeting RBP and TF networks could lead to novel therapeutic strategies.

Our multi-omics analysis revealed that PSME2 exhibits frequent copy number variations (CNVs) in breast cancer, and its variation status is significantly correlated with mRNA expression levels: it is markedly upregulated in samples with CNV amplification and significantly downregulated in samples with deletion (P < 0.01). Functional similarity analysis further supports the central role of PSME2 in the biological processes of breast cancer, with its functional score approaching or exceeding the significance threshold, indicating that this gene plays an important role in regulating tumor-related signaling pathways. Survival analysis results consistently show that high expression of PSME2 is significantly associated with poor patient prognosis, thereby establishing a comprehensive evidence chain of “CNVnncensiveentMRREF{5B826 From a mechanistic perspective, PSME2, as a component of the immunoproteasome, is known to be involved in antigen processing and the regulation of the immune microenvironment. By integrating single-cell RNA sequencing data, we identified that key genes exhibit differential expression patterns among immune cell subsets, such as PSMB8, which is predominantly expressed in macrophages, and BCL2, which is enriched in B lymphocytes. This further strengthens the inference that PSME2 may influence therapeutic response by modulating the tumor immune microenvironment. Therefore, changes in PSME2 expression not only reflect genomic structural variations but are also likely to impact immune recognition and tumor progression pathways, ultimately regulating the clinical outcomes of breast cancer. These findings provide multi-omics evidence for understanding the role of PSME2 in breast cancer and offer a theoretical basis for its potential as a therapeutic target or biomarker.

While our study provides significant insights into the molecular mechanisms underlying breast cancer, we acknowledge certain limitations. Firstly, this research primarily relies on bioinformatics analyses utilizing public databases such as TCGA and GEO, which lack detailed treatment histories and lifestyle information of individual patients. This limitation may restrict the comprehensiveness of our clinical correlation analysis. Regarding model validation, we observed a declining trend in the survival prediction capability of the model over time within the TCGA-BRCA cohort, with limited predictive efficacy for long-term prognosis (over five years). This time-dependent effect may arise from several factors: the absence of complete, long-term follow-up and event records in the cohort; the significant molecular subtype heterogeneity inherent in breast cancer, where some subtypes exhibit a higher risk of early recurrence, resulting in the model being more adept at short-term prognosis prediction; and variations in patient treatment regimens and time-varying risk changes that violate the proportional hazards assumption, all of which may impact long-term prediction performance. In the GEO validation cohort, due to limitations in data types and follow-up information, a systematic evaluation of this time-dependent effect remains unfeasible. In the drug sensitivity analysis section, this study considers the potential research and translational value of drug repurposing in cancer treatment. It not only focuses on drugs that have been widely utilized in clinical practice for breast cancer but also systematically includes and analyzes drugs approved for other cancer types that have yet to see widespread application in breast cancer. By investigating the sensitivity of these drugs, the study aims to provide novel research ideas and theoretical foundations for future targeted therapies and personalized medication in breast cancer, thereby broadening the scope of treatment strategies. It is important to note that the mechanisms of action and clinical efficacy of these drugs must be validated through further basic experiments and prospective clinical studies. Future research should integrate a wider range of clinical variables and incorporate multicenter validation to enhance the model’s generalizability and accuracy. It is crucial to emphasize that while the findings of this study reveal significant associations between Hedgehog-related genes and breast cancer prognosis, the immune microenvironment, and treatment sensitivity, these findings are primarily observational and aimed at proposing scientific hypotheses. They do not establish a causal relationship with the onset of breast cancer. Furthermore, discrepancies may exist between the results of enrichment analysis and the actual activation status of pathways. Therefore, while the model demonstrates clinical reference value for short-term risk stratification, its predictions regarding long-term risks should be interpreted with caution. Future research should prioritize the integration of multi-dimensional clinical variables to enhance the model’s universality and predictive accuracy. Particular emphasis should be placed on clarifying the specific mechanisms of these pathways in breast cancer through protein level detection, functional intervention experiments, and clinical sample validation. Additionally, utilizing *in vitro* and *in vivo* experimental model systems to validate the potential biological functions of these genes will be crucial for elucidating their molecular mechanisms.

In conclusion, our study contributes to the growing body of knowledge on breast cancer by elucidating the role of HRDEGs and associated pathways. Developing a robust prognostic model provides a foundation for future research into precision medicine strategies to improve patient outcomes through tailored therapeutic interventions. Continued exploration of the interactions between key genes and their pathways will advance our understanding of breast cancer biology and therapy.

## 5 Conclusions

This study developed a prognostic prediction model for breast cancer patients, integrating gene expression, immune response, drug response, and the Hedgehog (Hh) signaling pathway. Our findings highlight the significance of succinate in tumor metabolism and its potential role in therapeutic responses.

While this model enhances the understanding of survival factors in breast cancer, it has limitations, particularly the absence of personalized clinical data, which may compromise its accuracy. Future research should prioritize validating this model with larger and more detailed cohorts while also exploring the synergistic effects of key genes, including those related to succinate, on breast cancer progression and treatment responses. This approach aims to refine breast cancer management strategies and improve patient outcomes. Furthermore, this study developed a prognostic prediction model for breast cancer patients by integrating gene expression, immune response, drug response, and the Hedgehog (Hh) signaling pathway. Our findings underscore the importance of succinate in tumor metabolism and its potential role in therapeutic responses.

## Data Availability

The datasets presented in this study can be found in online repositories. The names of the repository/repositories and accession number(s) can be found in the article/**Supplementary Material**.

## Supplementary material

Supplementary Table 1 Genes Associated with Hedgehogs. This supplementary table provides information about genes associated with the Hedgehog pathway.

Supplementary Table 2 Differential Expression Table.

Supplementary Table 3 Network of mRNA-RBP Interactions (Nodes listed in **Supplementary Material**).

Supplementary Table 4 Nodes in the Network of mRNA-Transcription Factor Interactions (See **Supplementary Material** for details).

Supplementary Table 5 Immunological Correlations of 4 Genes.

Supplementary Table 6 Correlations of the HLA Family Genes with 4 Genes.

Supplementary Table 7 GSEA enrichment analysis based on Hs model.
