# Supplementary material for: Analyzing Hedgehog pathway-related genes: insights into breast cancer tumor microenvironment and prognostic implications
Source: Front Immunol. 2025 Dec 1;16:1649358. doi: 10.3389/fimmu.2025.1649358 (PMC12702891; doi:10.3389/fimmu.2025.1649358)
Supplement: Supplementary file 1 [file DataSheet1.docx]

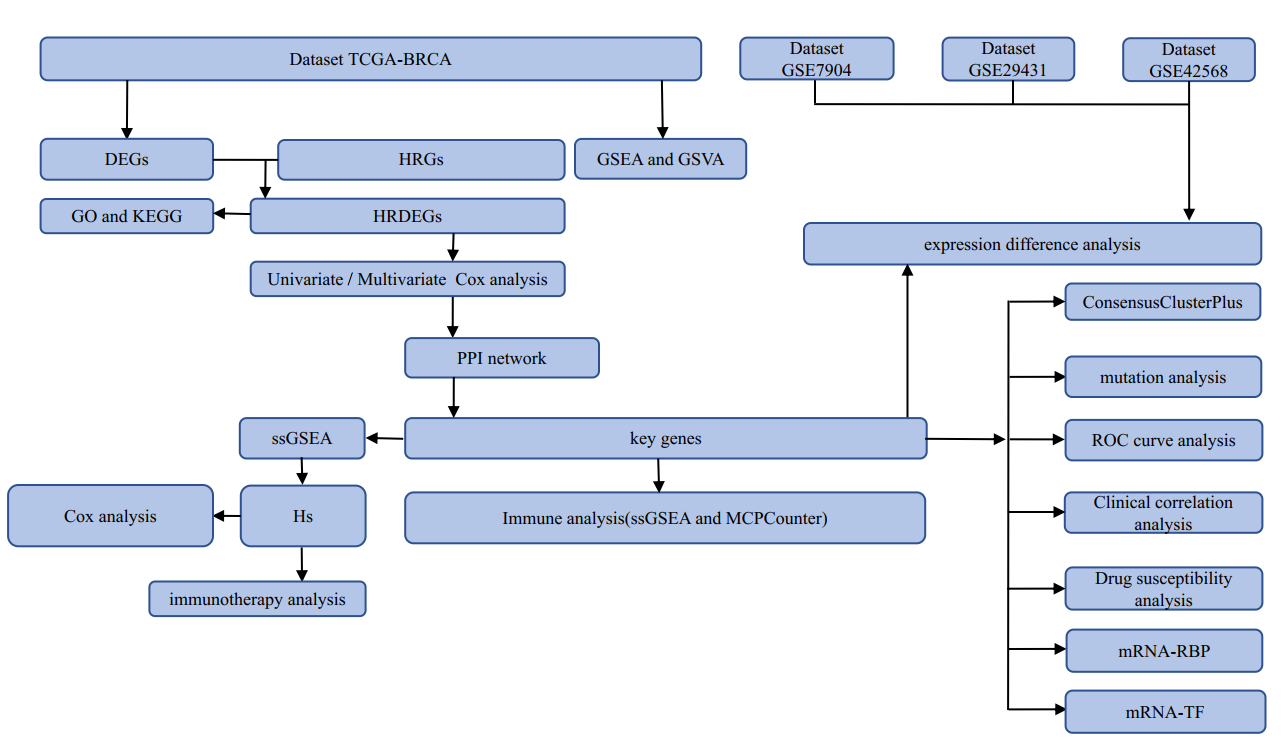


**Figure S1** Technical Roadmap

The diagram outlines the comprehensive workflow employed in this study. Starting with the TCGA-BRCA dataset, we performed an expression difference analysis. Gene Set Enrichment Analysis (GSEA) and Gene Set Variation Analysis (GSVA) were conducted using datasets GSE29431 and GSE7904. Differentially expressed genes (DEGs) were identified, followed by the identification of high-risk differentially expressed genes (HRDEGs) and high-risk genes (HRGs). Gene Ontology (GO) and KEGG pathway analyses were performed. Key genes were determined through univariate and multivariate Cox analyses. Immune analysis, including ssGSEA and MCPCounter, was performed, and subtypes were identified using ConsensusClusterPlus. Further analyses included drug susceptibility analysis, clinical correlation analysis, ROC curve analysis, mutation analysis, and PPI network construction. Immunotherapy analysis, mRNA-RBP interactions, and mRNA-TF interactions were also investigated.


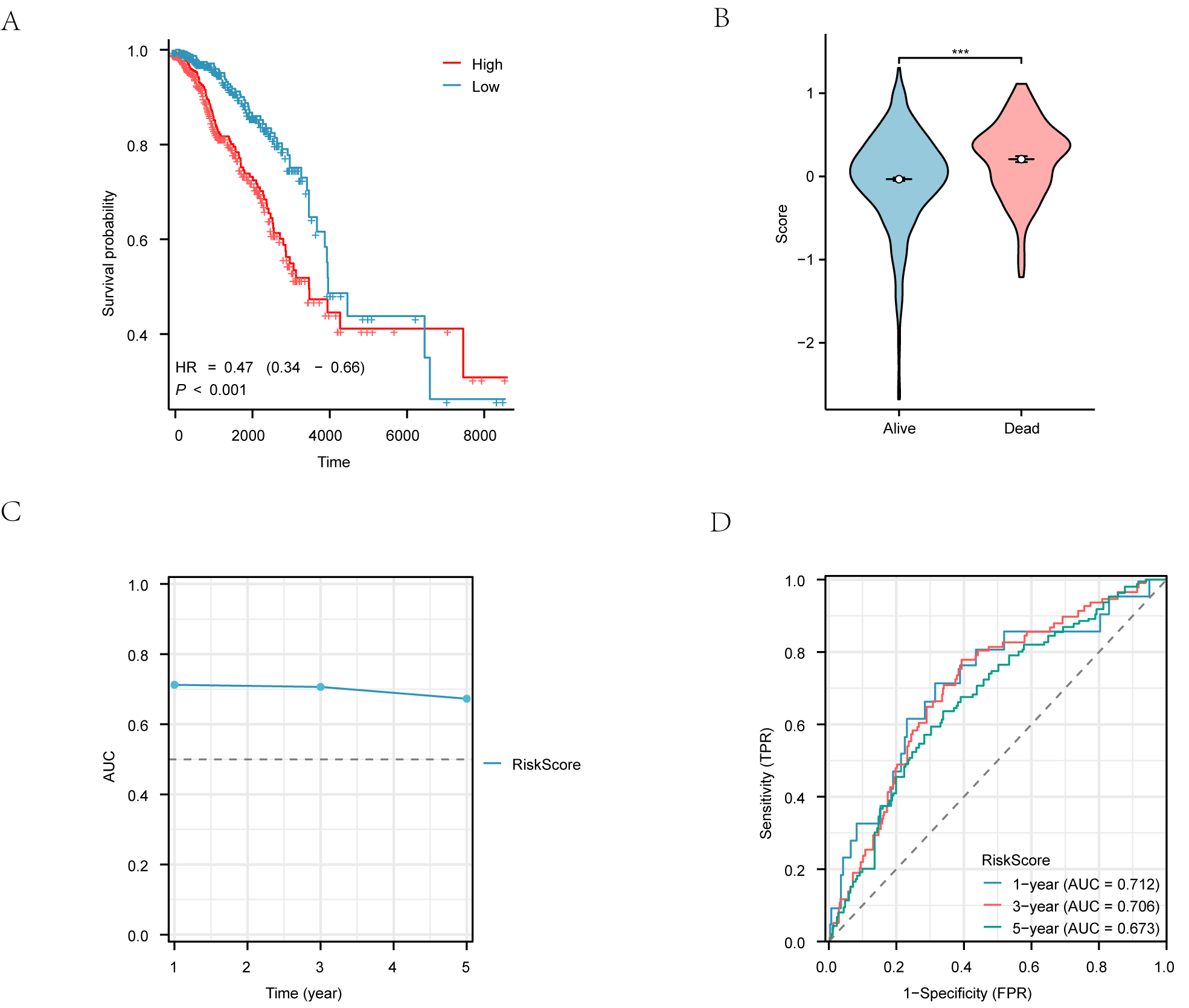


**Figure S2** Validation of the Cox regression model

A. Survival KM curve of prognostic Cox model RiskScore High and Low (High/Low) groups. B. Group comparison plot of prognostic Cox model (Dead/Alive) grouping. C. time-AUC curve of the prognostic Cox model RiskScore. D. time-ROC curve of prognostic Cox model RiskScore. P < 0.001, very significant difference. OS: Overall survival. KM: Kaplan-Meier. The risk factor map consists of two parts: 1. Risk grouping: the RiskScore predicted by the Cox regression prognostic model is grouped by the median. 2. Survival outcomes: Dot plot was used to show the survival time and survival outcomes of the breast cancer dataset TCGA-BRCA clinical samples.


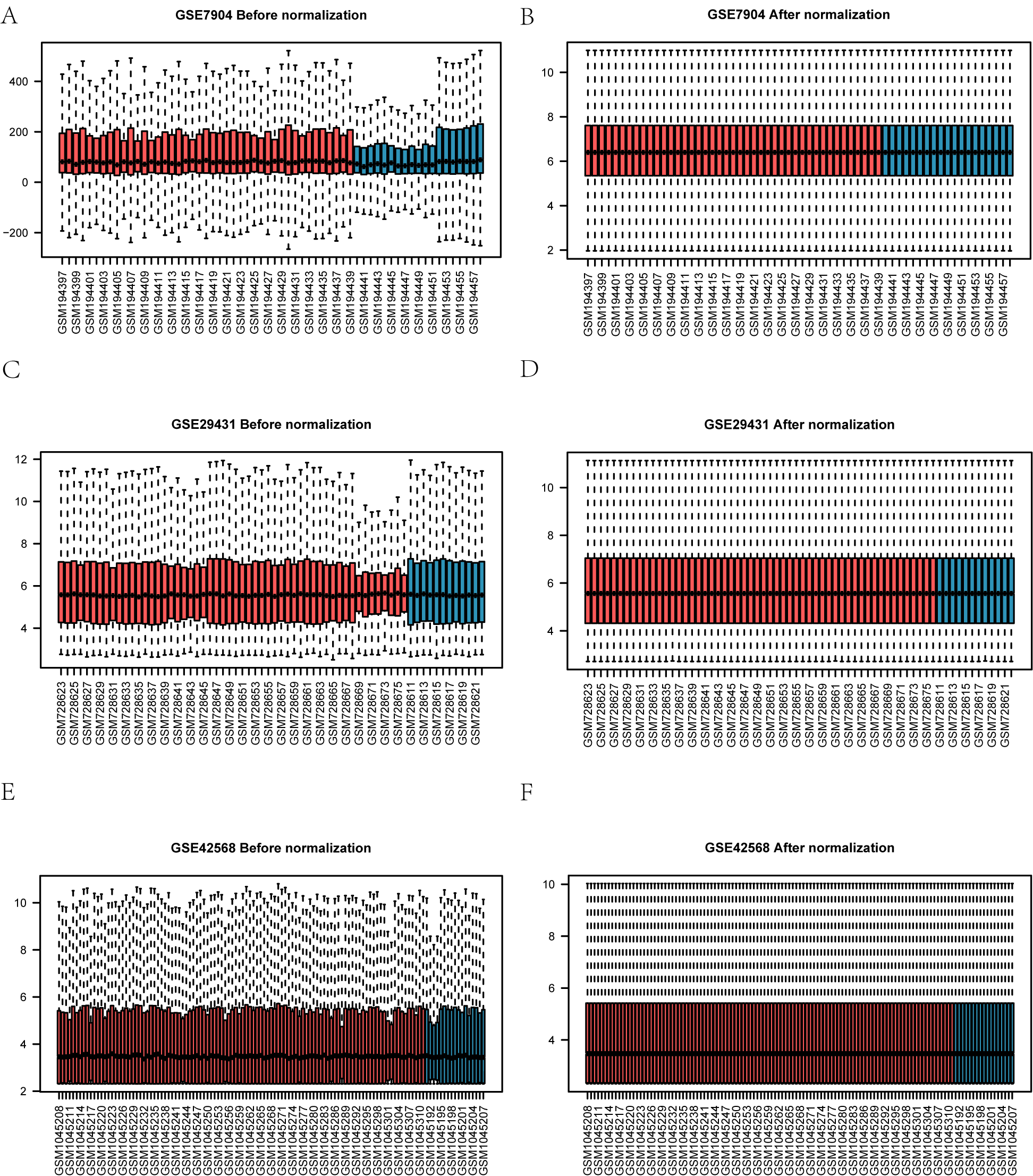


**Figure S3** Standardizing Datasets for Breast Cancer

This figure demonstrates the process of standardizing breast cancer datasets, including GSE7904, GSE29431, and GSE42568. The data normalization using the R package limma reveals consistent patterns among the samples in each dataset before (A, C, E) and after (B, D, F) standardization.


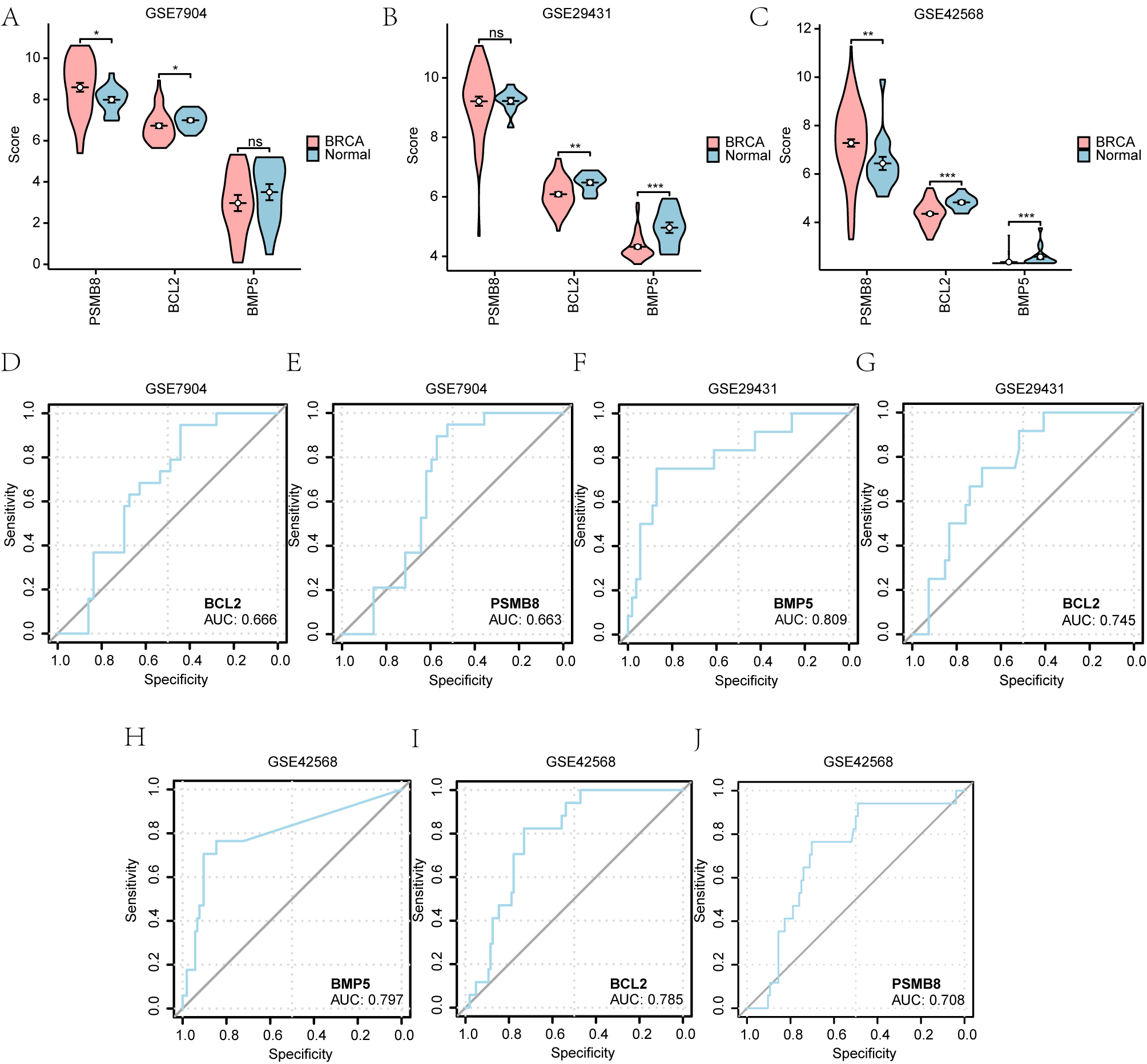


**Figure S4** Examining Key Genes in the GEO Datasets A-C. Group comparison results of key genes among different groups (BRCA/Normal) in datasets GSE7904 (A), GSE29431 (B) and GSE42568 (C) are shown. The blue color represents the Normal group and the red color represents the BRCA group. D-e. ROC curve analysis of key genes BCL2 (D) and PSMB8 (E) in dataset GSE7904. F-g. ROC curve analysis of key genes BMP5 (F) and BCL2 (G) in dataset GSE29431. H-j. ROC curve analysis of key genes BMP5 (H), BCL2 (I), and PSMB8 (J) in dataset GSE42568.


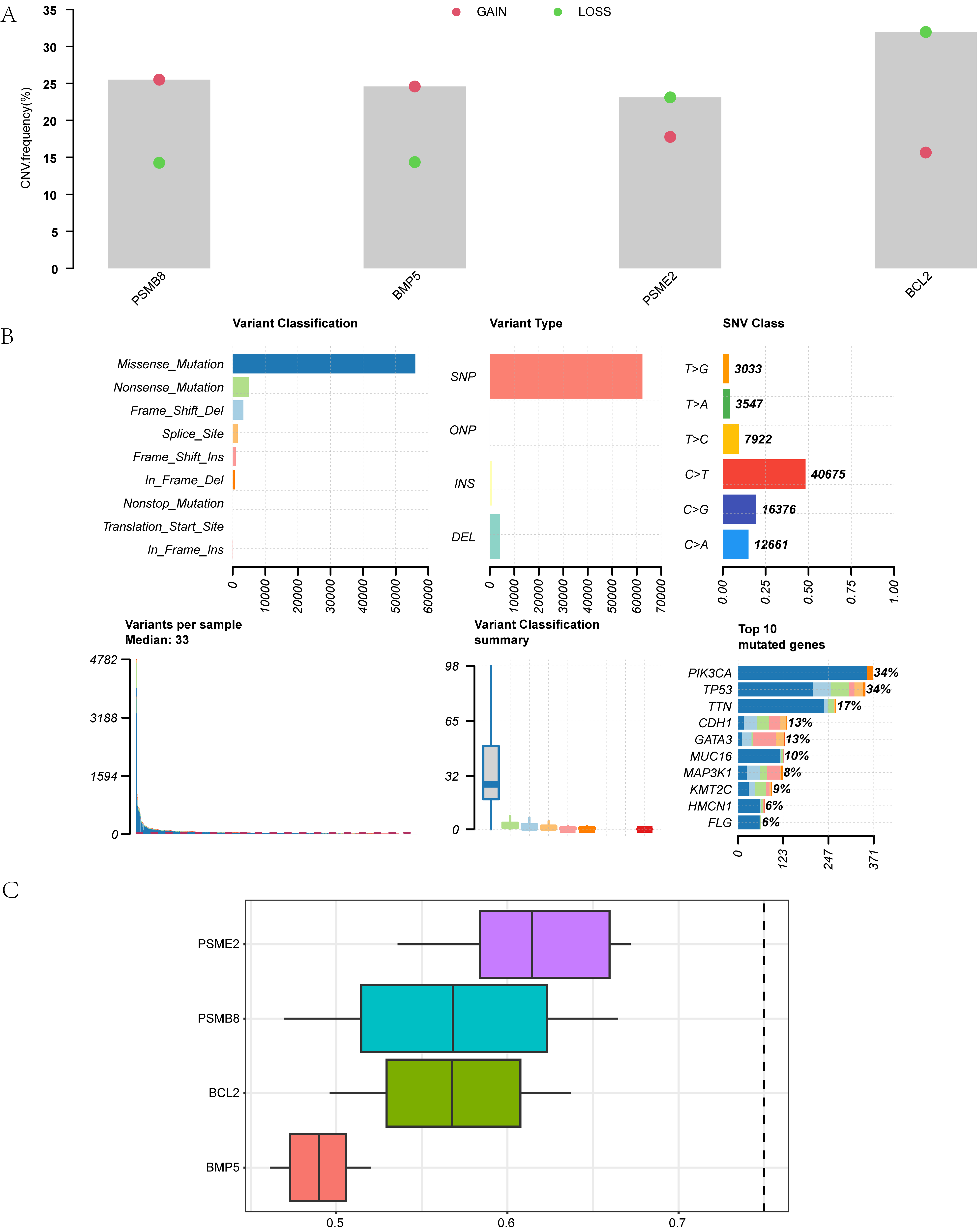


**Figure S5** CNV, SNP, and Friends Analysis

This figure combines information on Copy Number Variations (CNV)(A), Single Nucleotide Polymorphisms (SNPs) (B), and Functional Similarity (Friends) analysis (C) to gain insights into breast cancer (BRCA) using the TCGA-BRCA dataset. It provides a comprehensive view of genetic alterations, mutations, and the influence of specific genes on the disease. The data is crucial for understanding the molecular underpinnings of breast cancer and its clinical implications.


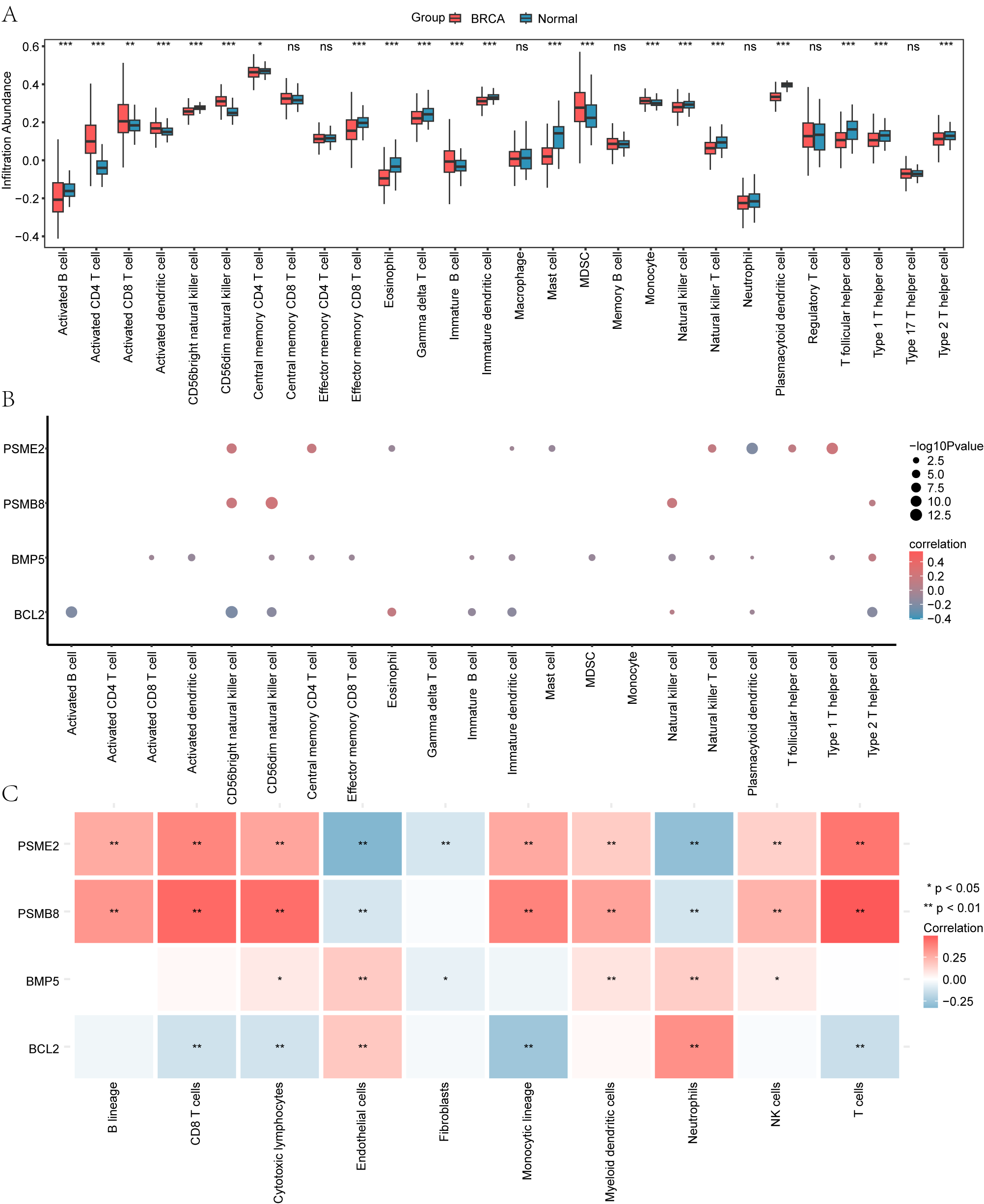


**Figure S6** Immune infiltration analysis (ssGSEA and MCPCounter)

(A)The ssGSEA algorithm was used to compare 28 immune cell types in the TCGA-BRCA dataset, specifically emphasizing distinct groups (normal/BRCA). (B)The results of the correlation analysis using the ssGSEA algorithm are shown in a heatmap illustrating the infiltration abundance of 21 immune cells with a p-value below 0.05. (C)The MCPCounter algorithm computed the correlation analysis findings among essential genes and the level of immune cell infiltration, which were subsequently represented as a heatmap.


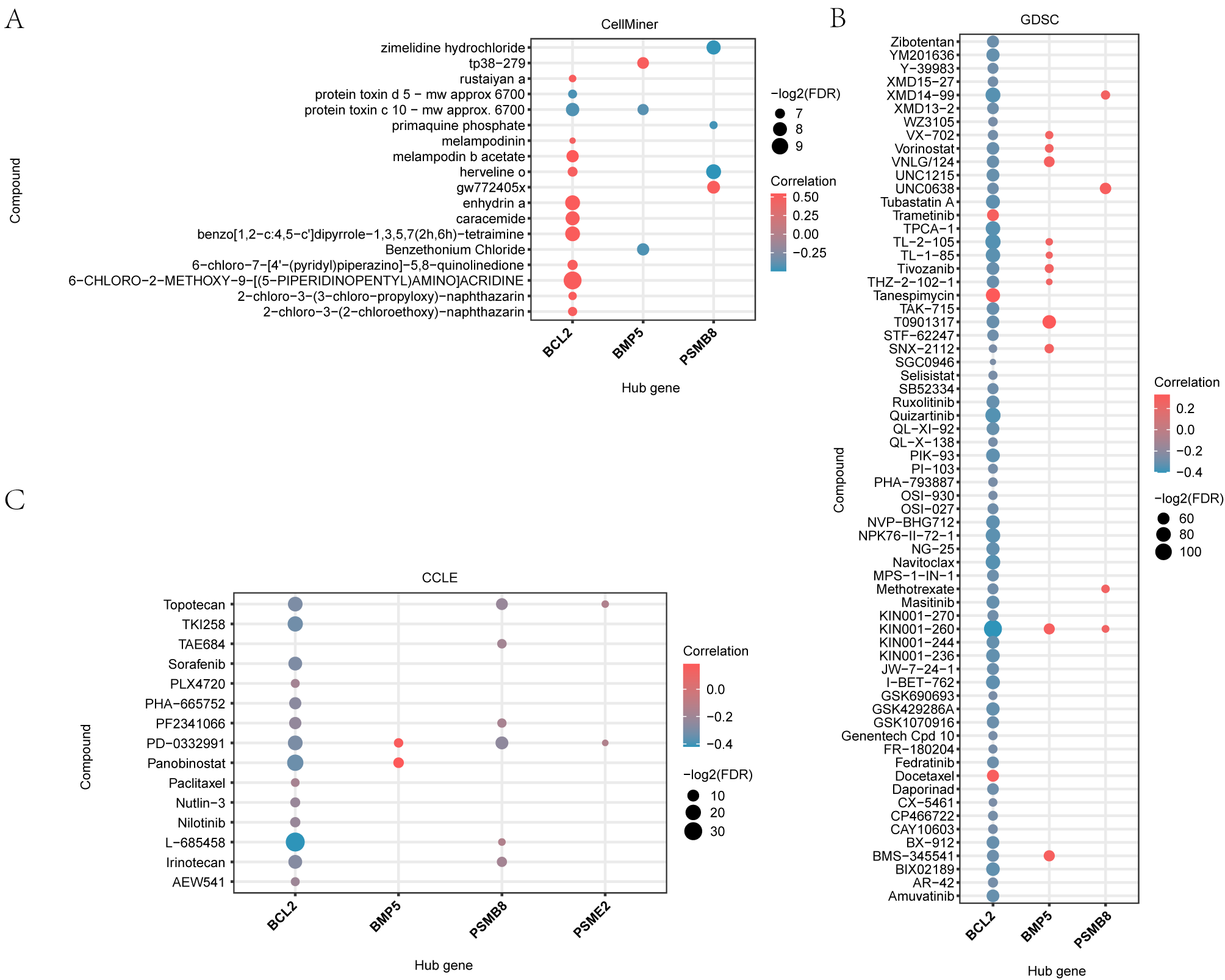


**Figure S7** Drug Sensitivity Analysis Results

This section presents the outcomes of drug sensitivity analysis for significant genes in the CellMiner (A), GDSC (B), and CCLE (C) databases. The visualizations illustrate correlations, with red circles indicating positive links between genes and immune cell infiltration. As the circle size increases, the strength of the correlation intensifies. Conversely, blue circles, whose size also increases, symbolize negative correlations between genes and the abundance of infiltrating immune cells. The acronyms GDSC and CCLE stand for Genomics of Drug Sensitivity in Cancer and the Cancer Cell Line Encyclopedia, respectively.


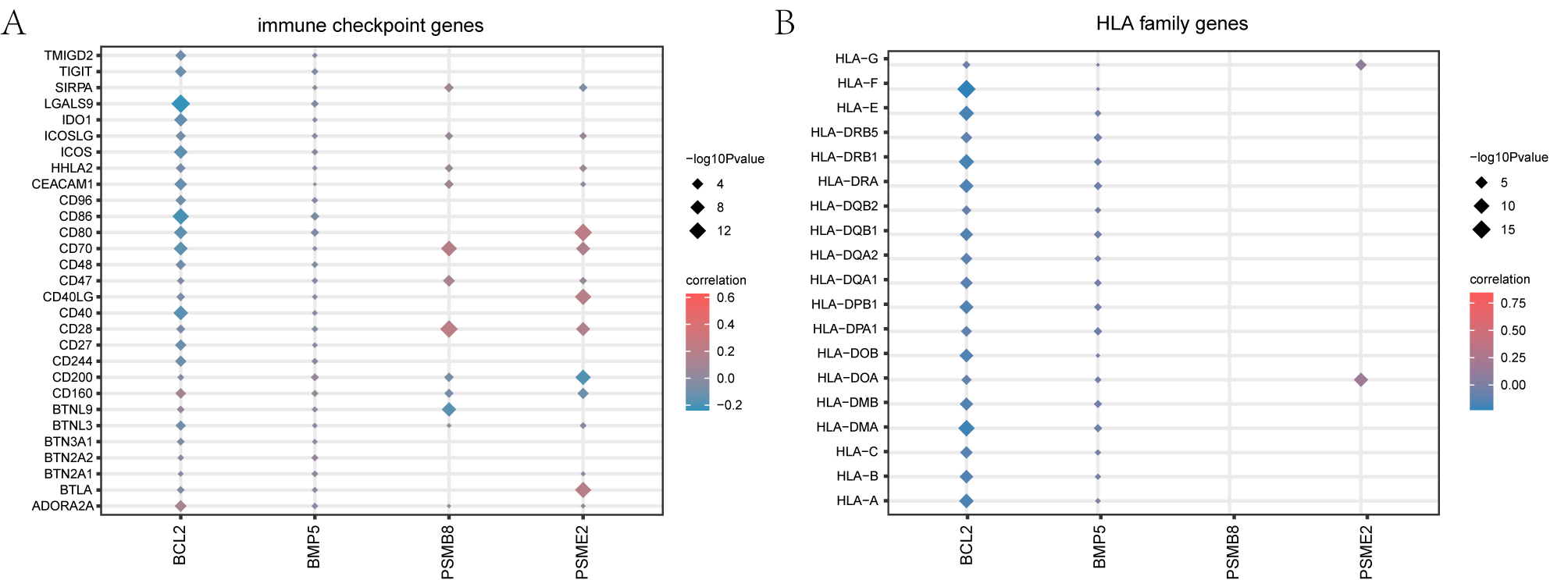


**Figure S8** Correlation Analysis and Disease Subtype Construction

1. Heatmap of the correlation between key genes and immune checkpoints is shown. B. Heat map of correlation between key genes and HLA family genes.


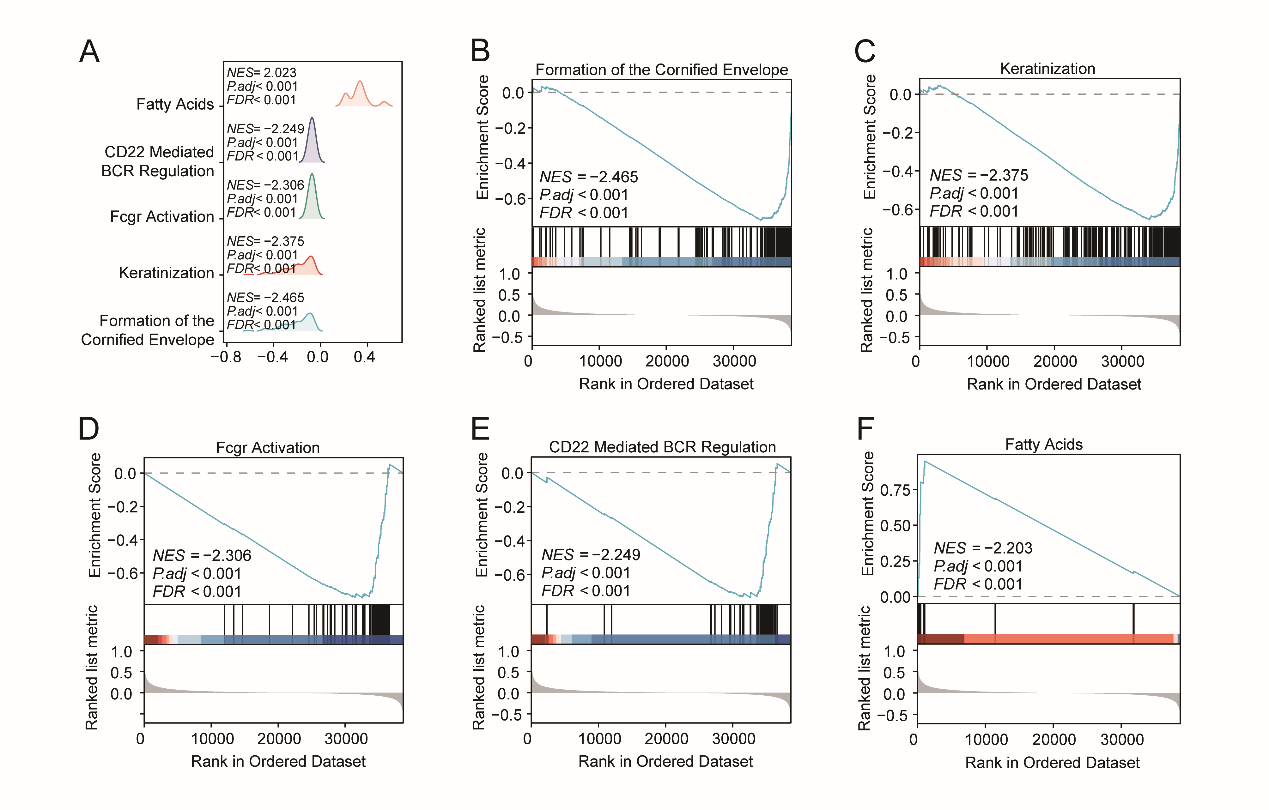
 **Figure S9** GSEA Enrichment Analysis Results for TCGA-BRCA Dataset

A. The GSEA enrichment analysis of the data set TCGA-BRCA mainly contains 5 main biological characteristics. The analysis focused on five prominent biological characteristics, namely the Cornified Envelope formation (B), Keratinization (C), Fcgr Activation (D), CD22 Mediated BCR Regulation (E), and Fatty Acid metabolism (F) pathways, along with various others. GSEA, which stands for Gene Set Enrichment Analysis, is a method used to explore gene set enrichment.


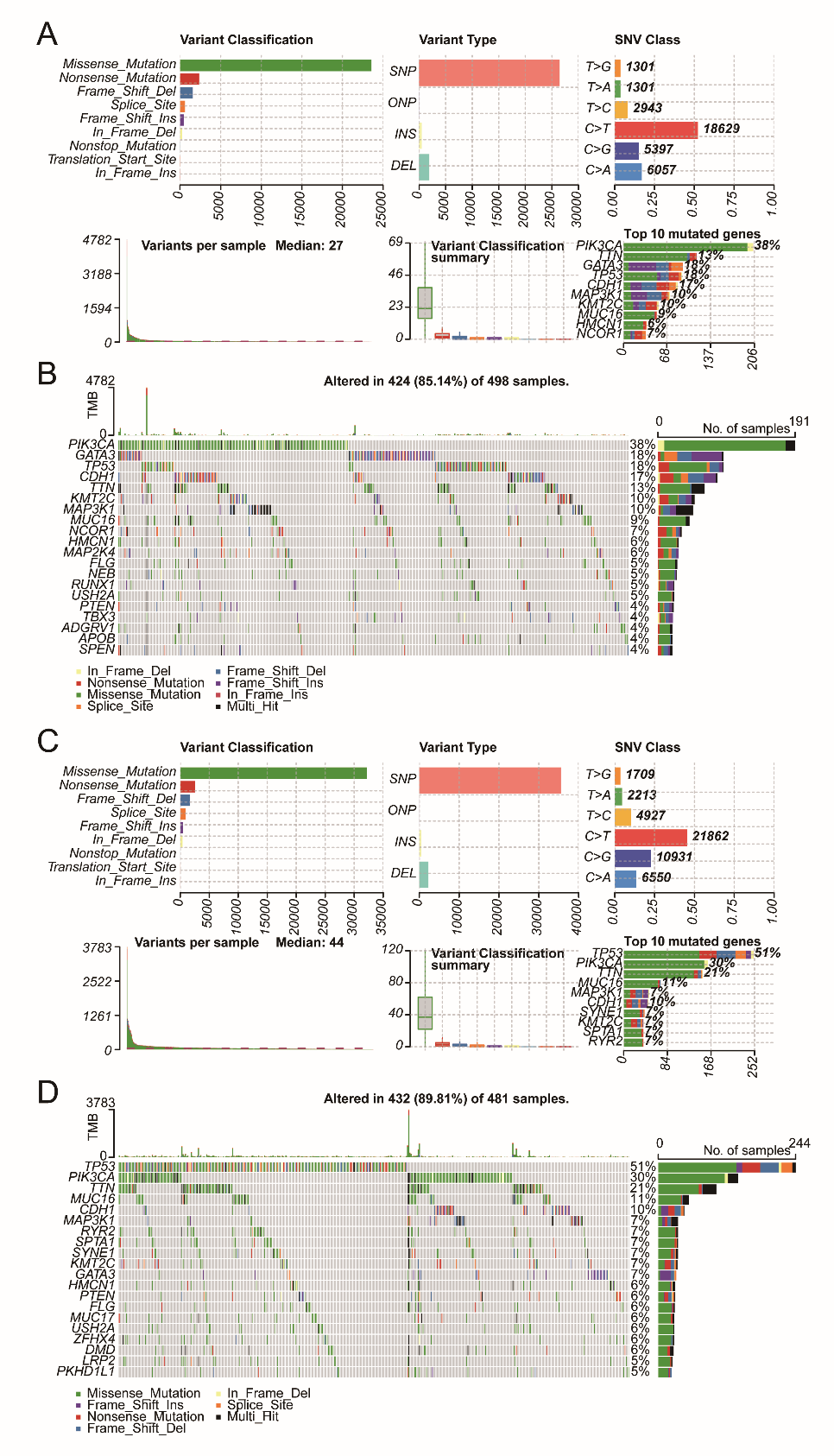


**Figure S10** Comparative SNP Analysis in TCGA-BRCA Dataset (A)Display somatic mutations (SNP) in high HR score group samples. (B)Waterfall chart showcasing the top 20 genes with the highest number of somatic mutations in high HR score group samples. (C)Display somatic mutations (SNP) in low HR score group samples. (D)Waterfall chart showcasing the top 20 genes with the most somatic mutations in low HR score group samples.


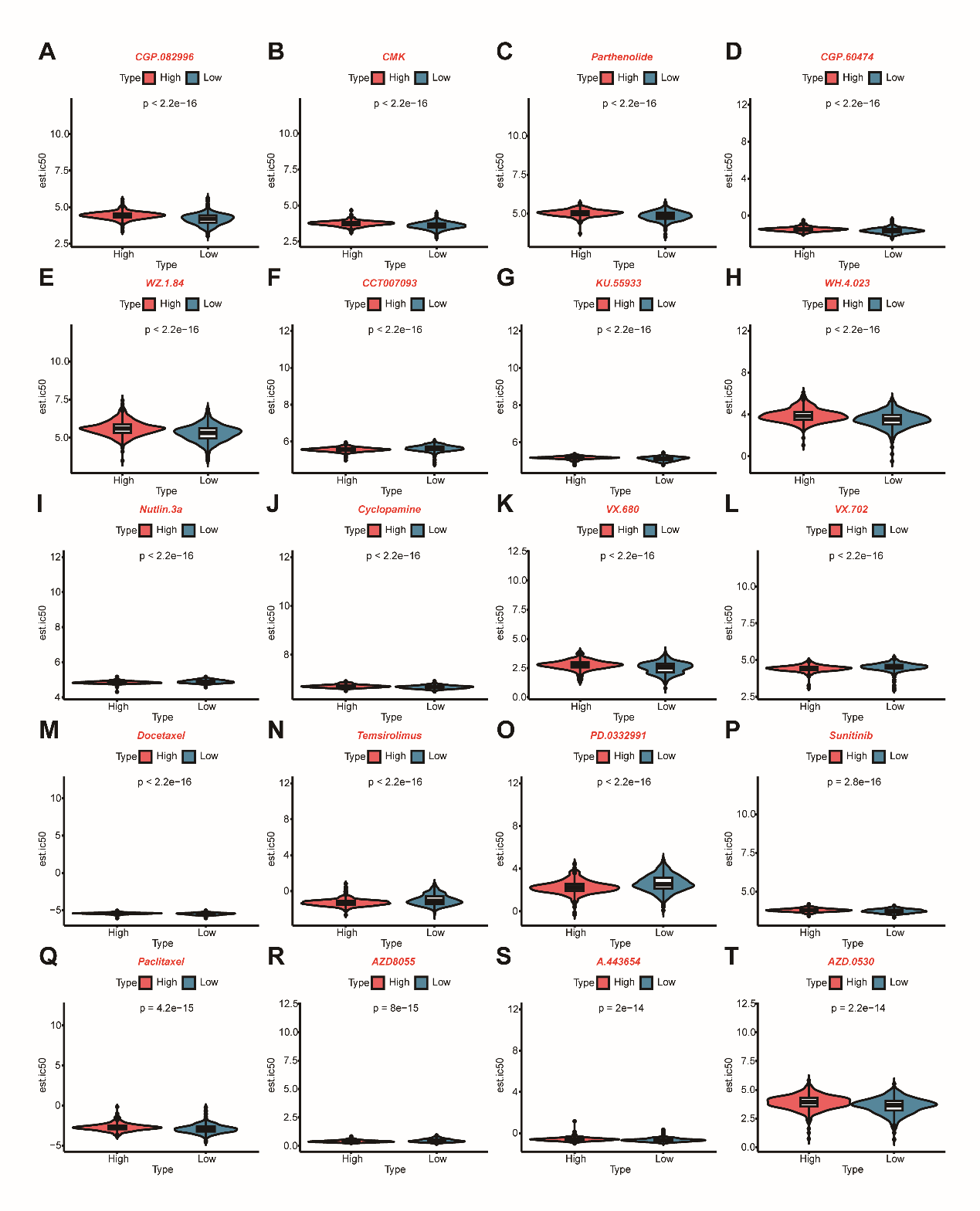


**Figure S11** Drug Sensitivity Analysis in TCGA- RCA Dataset

The GDSC database classifies HR scores concerning drugs such as CGP.082996 (A), CMK (B), parthenolide (C), CGP,60474 (D), WZ.1.84 (E), CCT007093 (F), KU.55933 (G), WH.4.023 (H), Nutlin.3a (I), cyclopamine (J),and VX.680 (K). In the visualization, red represents the high HR score group, while blue designates the low HR score group. GDSC stands for Genomics of Drug Sensitivity in Cancer.


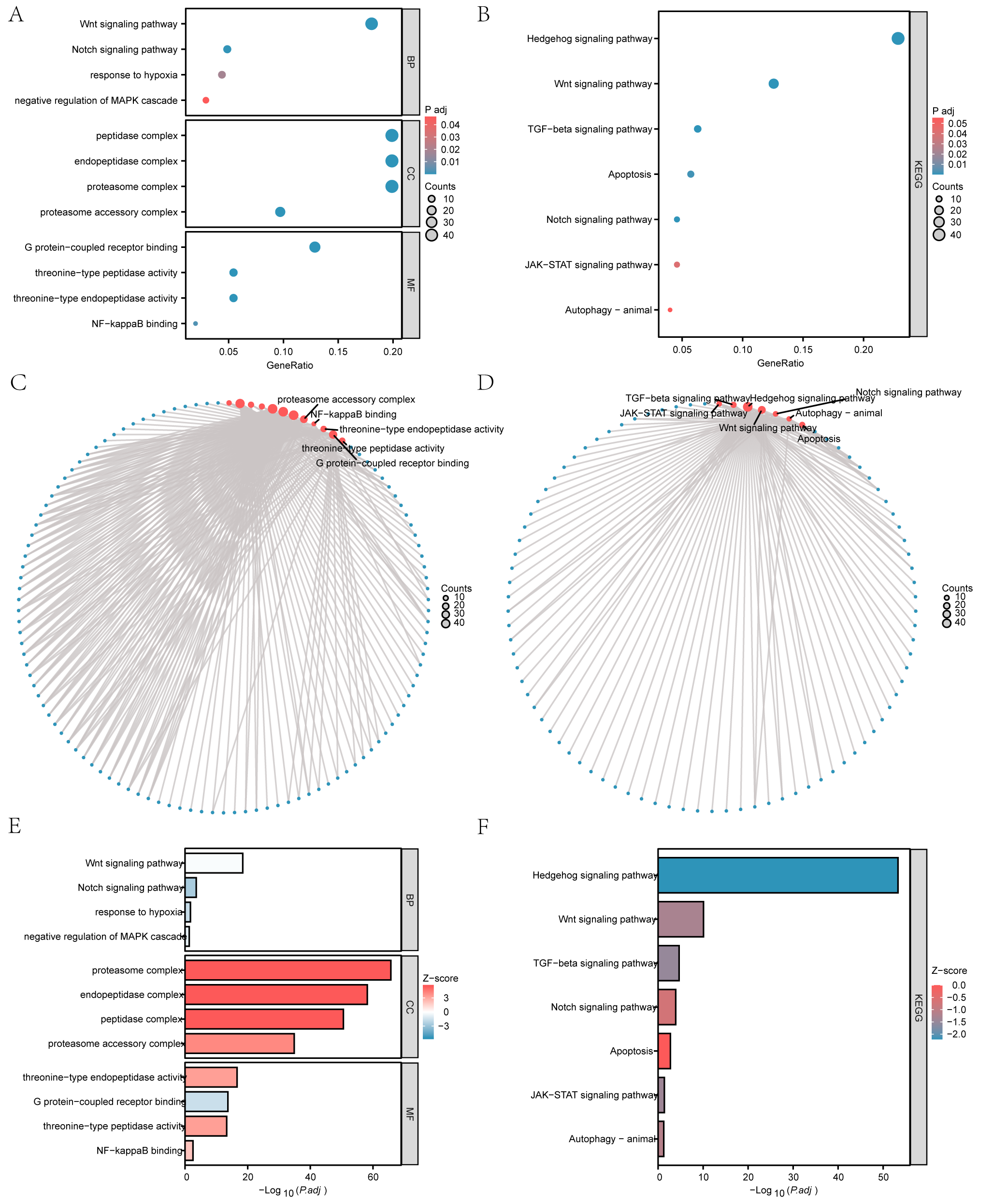


**Figure S12** Functional and Pathway Enrichment Analysis **(A):** The bubble plot provides an overview of Highly Differentially Expressed Genes (HRDEGs) and their enrichment in biological processes (BP) as part of Gene Ontology (GO) analysis. **(B):** The bubble plot displays the HRDEGs and their enrichment in KEGG pathways. **(C):** According to GO analysis, the circular network diagram illustrates the relationships and connections of HRDEGs in enriched biological processes. **(D):** The circular network diagram represents the relationships and connections of HRDEGs in enriched KEGG pathways. **(E):** The bar chart visually presents the outcomes of GO functional enrichment analysis for HRDEGs, focusing on biological processes (BP), cellular components (CC), and molecular functions (MF). **(F):** The bar chart depicts the KEGG pathway enrichment analysis results for HRDEGs.


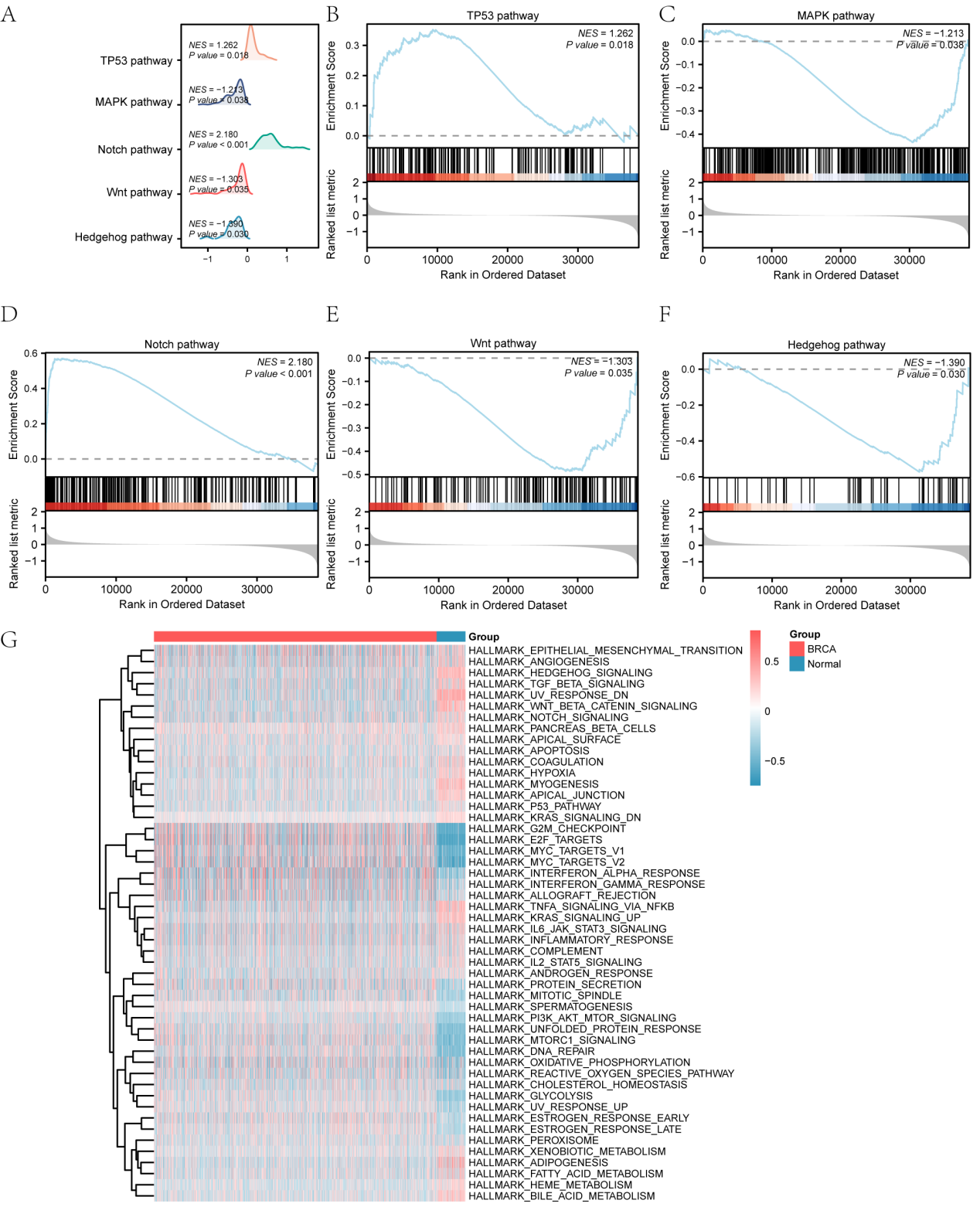


**Figure S13** Enrichment analysis of the TCGA-BRCA dataset using GSEA and GSVA. (A) GSEA was utilized to discover the top five significant biological characteristics, revealing substantial enrichment in pathways such as TP53 (B), MAPK (C), Notch (D), Wnt (E), and Hedgehog (F), along with additional pathways. (G) GSVA analysis in dataset TCGA-BRCA. The TCGA-BRCA dataset was analyzed using both GSEA and GSVA, where Normal breast cancer samples were represented in blue and breast cancer patient samples in red. The primary criteria for screening enrichment in GSEA and GSVA were a p-value below 0.05 and an FDR value (q-value) below 0.25.


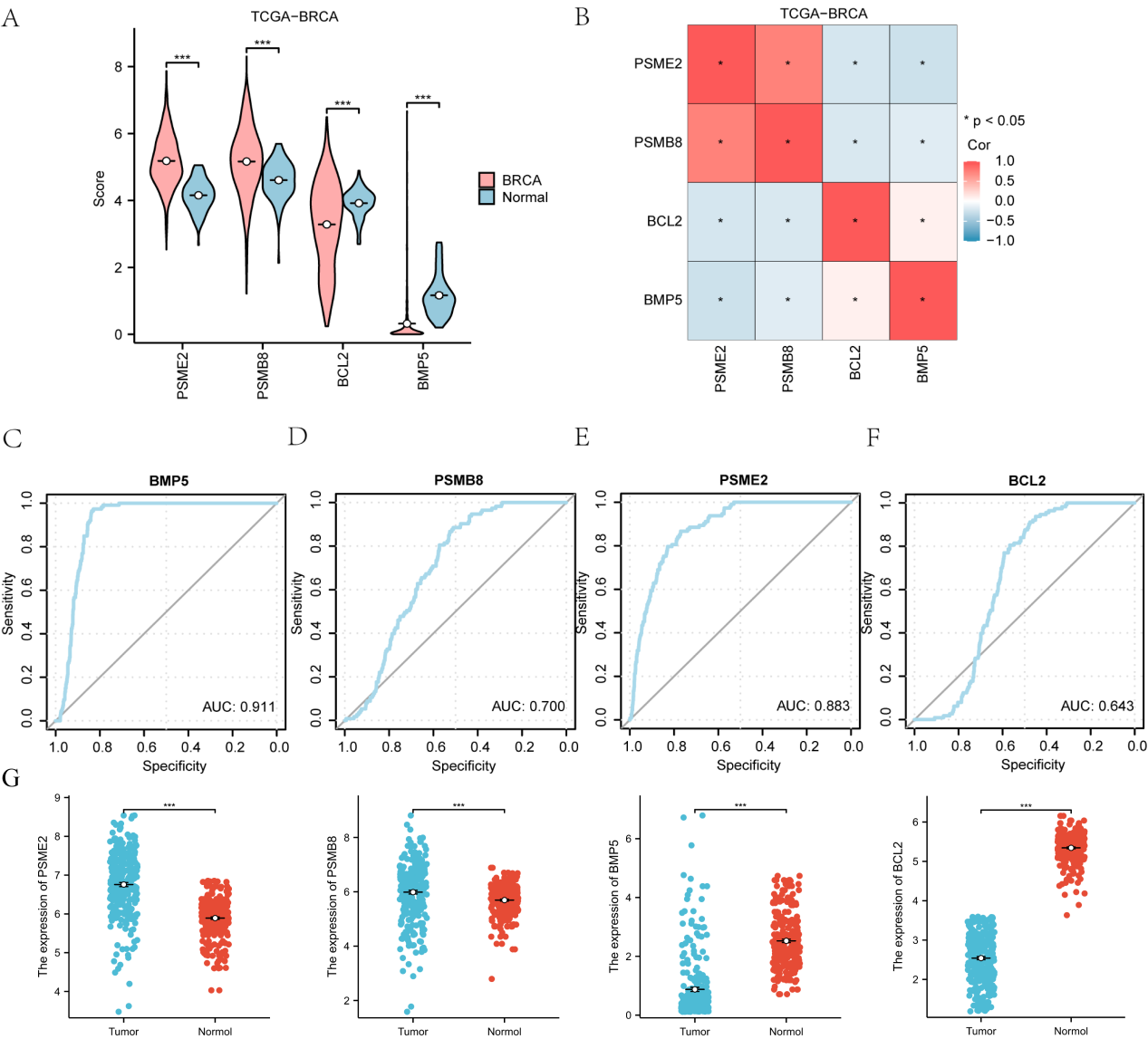


**Figure S14** Expression of Key Genes in TCGA-BRCA Breast Cancer Dataset

In Figure 9, key genes are examined within the TCGA-BRCA breast cancer dataset. This figure compares these key genes between BRCA and normal groups, represented in red and blue (A), respectively. The heat map visualizes the correlation among the significant genes in the TCGA-BRCA breast cancer dataset (B). ROC curve analysis was conducted for the important genes BMP5 (C), PSMB8 (D), PSME2 (E), and BCL2 (F) within the TCGA-BRCA dataset. The diagnostic performance of these genes is assessed based on the area under the curve (AUC). AUC values above 0.9 indicate a high level of precision, values between 0.7 and 0.9 signify moderate precision, and values between 0.5 and 0.7 suggest relatively low precision. PCR results revealed that PSME2 and PSMB8 were highly expressed in cancer tissues, while BMP5 and BCL2 were highly expressed in normal tissues (G).
